# Molecular Mechanism of Different Rooting Capacity between Two Clones of *Taxodium hybrid* ‘Zhongshanshan’

**DOI:** 10.3390/ijms25042427

**Published:** 2024-02-19

**Authors:** Jiaqi Liu, Lei Xuan, Chaoguang Yu, Jianfeng Hua, Ziyang Wang, Yunlong Yin, Zhiquan Wang

**Affiliations:** 1 Institute of Botany, Jiangsu Province and Chinese Academy of Sciences, Nanjing 210014, China; 15129740979@163.com (J.L.); 13851991791@163.com (L.X.); yuchaoguang168@cnbg.net (C.Y.); jfhua2009@gmail.com (J.H.); wangziyang@cnbg.net (Z.W.); 2 Jiangsu Key Laboratory for the Research and Utilization of Plant Resources, Nanjing 210014, China

**Keywords:** *Taxodium hybrid* ‘Zhongshanshan’, adventitious root formation, cutting propagation, transcriptome, gene function identification

## Abstract

The conifer *Taxodium hybrid* ‘Zhongshanshan’ (*T. hybrid* ‘Zhongshanshan’) is characterized by rapid growth, strong stress resistance, and high ornamental value and has significant potential for use in afforestation, landscaping, and wood production. The main method of propagating *T. hybrid* ‘Zhongshanshan’ is tender branch cutting, but the cutting rooting abilities of different *T. hybrid* ‘Zhongshanshan’ clones differ significantly. To explore the causes of rooting ability differences at a molecular level, we analyzed the transcriptome data of cutting base and root tissues of *T. hybrid* ‘Zhongshanshan 149’ with a rooting rate of less than 5% and *T. hybrid* ‘Zhongshanshan 118’ with rooting rate greater than 60%, at the developmental time points in this study. The results indicated that differentially expressed genes between the two clones were mainly associated with copper ion binding, peroxidase, and oxidoreductase activity, response to oxidative stress, phenylpropanoid and flavonoid biosynthesis, and plant hormone signal transduction, among others. The expression pattern of *ThAP2* was different throughout the development of the adventitive roots of the two clone cuttings. Therefore, this gene was selected for further study. It was shown that *ThAP2* was a nuclear-localized transcription factor and demonstrated a positive feedback effect on rooting in transgenic *Nicotiana benthamiana* cuttings. Thus, the results of this study explain the molecular mechanism of cutting rooting and provide candidate gene resources for developing genetic breeding strategies for optimizing superior clones of *T. hybrid* ‘Zhongshanshan’.

## 1. Introduction

Asexual reproduction is often used for the propagation of forestry trees; it not only avoids the segregation of characteristics during seed propagation and fixes heterosis resulting from hybridization but also produces stable and consistent genetic populations to facilitate mass production and management [1]. Vegetative organs, such as roots, stems, and leaves, can be used for asexual reproduction via methods such as cutting, grafting, and layering [2,3]. Of these, using cuttings is perhaps the simplest, resulting in rapid seedling formation, strong root systems, strong growth potential and stress resistance, and low cost. Therefore, propagation via cutting has an important role in forestry and horticulture, such as for poplars [4,5], willows [6], black locusts [7], and other nursery species.

Cuttings have adventitious buds, which makes them suitable material for vegetative propagation. Under appropriate conditions, cuttings grow at both ends, forming a crown upward and differentiating downward to form adventitious roots (AR), resulting in an independent plant [2]. Roots absorb and transport water and nutrients from the soil for plant nutrition and reproductive growth. The root system also has a fixation and support function, which keeps the plant upright and protects it from being knocked over by strong winds and rain. Therefore, AR formation is key to propagating seedlings from cuttings [8].

*Taxodium hybrid* ‘Zhongshanshan’ (*T. hybrid* ‘Zhongshanshan’) is the general name for hybrids of *Taxodium*, developed by the Institute of Botany, Jiangsu Province and Chinese Academy of Sciences, Nanjing Jiangsu, China. *T. hybrid* ‘Zhongshanshan’ shows rapid growth, strong stress resistance, and high ornamental value and, thus, has significant potential for use in afforestation, landscaping, and wood production. Varieties of *T. hybrid* ‘Zhongshanshan’ are widely used in the afforestation of wetlands, beaches, and plains in the Yangtze River basin and southeast coastal areas of China. Years of breeding work focusing on *T. distichum* × *T. mucronatum*, (*T. distichum × T. mucronatum*) × *T. mucronatum*, and *T. mucronatum × T. distichum* have resulted in improved clones. Their propagation is mainly achieved using cuttings [3]. However, such propagation has resulted in significant differences in the rooting ability of different clones, limiting their application.

*T. hybrid* ‘Zhongshanshan 118’ and *T. hybrid* ‘Zhongshanshan 149’ are both second-stage clones. However, field experiments revealed differences in their rooting ability. To explore the causes of rooting ability differences at the molecular level, in this study, we conducted transcriptome analysis of cutting base and root tissues of *T. hybrid* ‘Zhongshanshan 118’ and *T. hybrid* ‘Zhongshanshan 149’ to identify the candidate genes related to AR formation. *ThAP2* was identified based on the clustering and enrichment analysis and then demonstrated a positive effect on rooting in transgenic *Nicotiana benthamiana (N. benthamiana*) cuttings. This study will provide useful information and potential genetic resources for the molecular breeding of *T. hybrid* ‘Zhongshanshan’.

## 2. Results

### 2.1. Rooting Observation and Statistical Results

**Figure 1:**
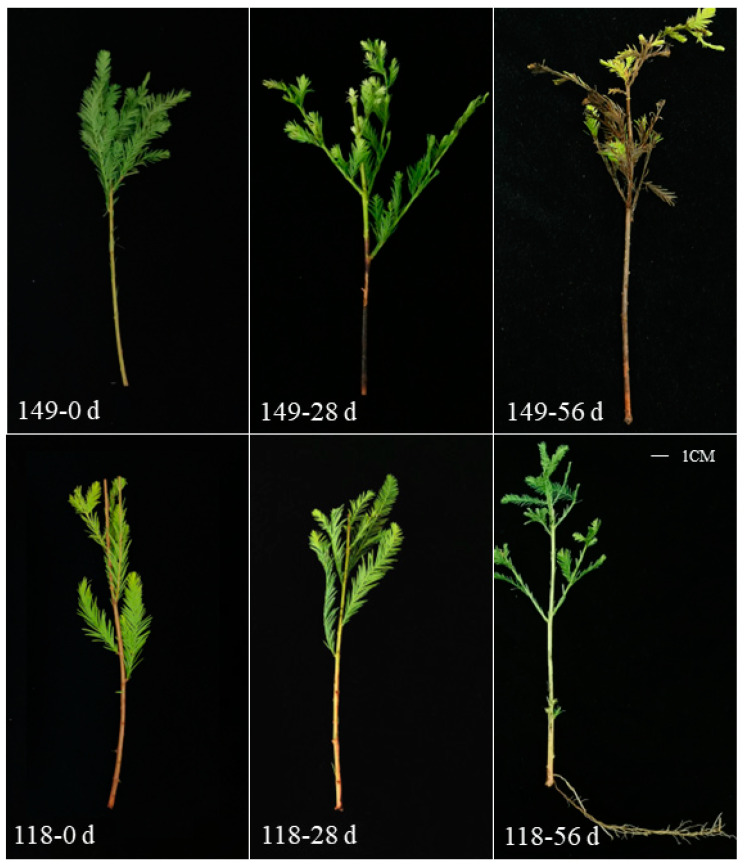
AR formation of *T. hybrid* ‘Zhongshanshan 149’ and *T. hybrid* ‘Zhongshanshan 118’ cuttings at different developmental time points. Three time points were used in the study. The first time point for sample was 0 day (The samples were marked as 149–0 d and 118–0 d), the second time point was 28 days after cutting (149–28 d and 118–28 d), and the third time point was 56 days after cutting (149–56 d and 118–56 d).

Callus formation rate and rooting rate were observed and calculated every 7 days after cutting, which showed the two clones’ different rooting abilities after cutting (Table 1, Figure 1). At 28 days after cutting, the callus ratio of *T. hybrid* ‘Zhongshanshan 118’ was 84.42%, which was significantly different from that of *T. hybrid* ‘Zhongshanshan 149’. At this time, a lot of cuttings of *T. hybrid* ‘Zhongshanshan 149’ were turning black at the base. At 56 days after cutting, the rooting rate of *T. hybrid* ‘Zhongshanshan 118’ was 62.39%, whereas that of *T. hybrid* ‘Zhongshanshan 149’ was much lower. Most of the cuttings of *T. hybrid* ‘Zhongshanshan 149’ were still black and began to wither and die. Thus, three time points were used in the study. The first time point for the sample was 0 day (The samples were marked as 149–0 d and 118–0 d), the second time point was 28 days after cutting (149–28 d and 118–28 d), and the third time point was 56 days after cutting (149–56 d and 118–56 d).

### 2.2. Summary of Transcriptome Data

**Figure 2:**
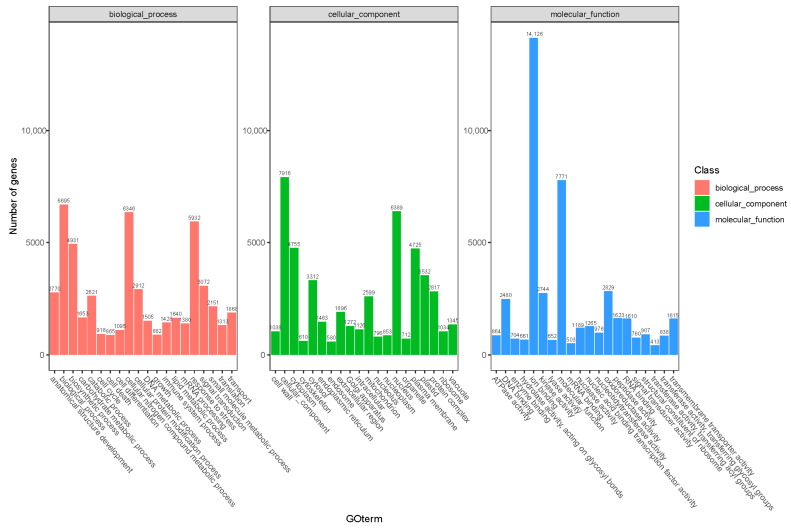
GO annotation of the combined transcriptome data from *T. hybrid* ‘Zhongshanshan 149’ and *T. hybrid* ‘Zhongshanshan 118’.

**Figure 3:**
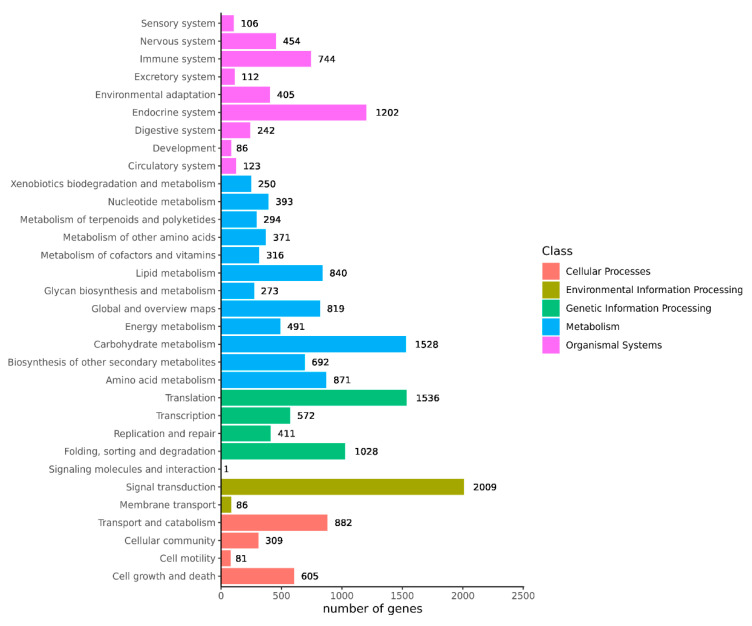
KEGG annotation of the combined transcriptome data from *T. hybrid* ‘Zhongshanshan 149’ and *T. hybrid* ‘Zhongshanshan 118’.

Transcriptome sequencing was performed on the cuttings to obtain an overview of the gene expression profiles of the two types of *T. hybrid* ‘Zhongshanshan’ after cutting. After removing low-quality adapter/primer contaminants and duplicated reads, clean reads were generated (Table S1). Ultimately, 41,109 non-redundant unigenes were obtained (Table 2). To obtain comprehensive gene function information, the unigenes were annotated using seven databases: the UniProt Knowledgebase (UniProt), Kyoto Encyclopedia of Genes and Genomes (KEGG), Gene Ontology (GO), NCBI non-redundant protein sequences (Nr) and others (Figure S1, Table S2). In the GO database, 6346 and 5932 genes were annotated at the biological process (BP) level in the cellular nitrogen compound metabolic process and response to stress categories, respectively. At the cellular component (CC) level, 6389 genes were significantly annotated in the nuclear category, whereas, at the molecular function (MF) level, 14,126 and 7771 genes were significantly annotated in the ion binding and molecular function categories, respectively (Figure 2). Based on the KEGG analysis, 2009, 1536, 1528, and 1202 genes were annotated significantly in the signal transduction, translation, carbohydrate metabolism, and endocrine system categories, respectively (Figure 3).

### 2.3. Transcriptome Comparisons

**Figure 4:**
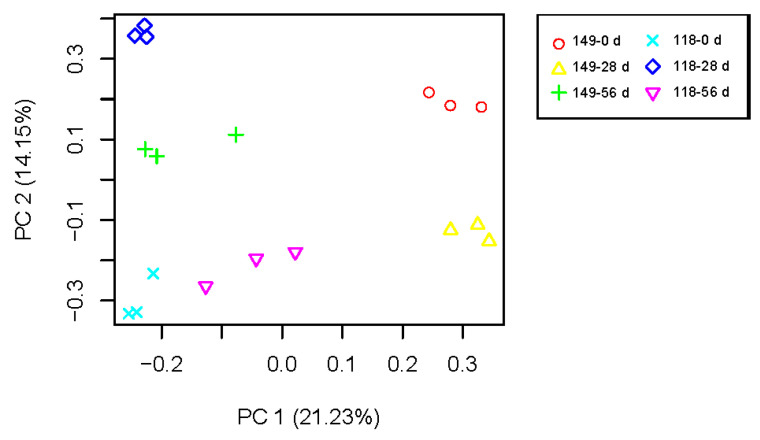
PCA of mRNA populations of samples of *T. hybrid* ‘Zhongshanshan 149’ and *T. hybrid* ‘Zhongshanshan 118’.

To understand AR formation in the two clones of *T. hybrid* ‘Zhongshanshan’, the transcriptome data were subjected to PCA (Figure 4). The data points of biological duplication clustered approximately together, and different samples could be clearly distinguished, indicating the repeatability of sequencing results is good. There were significant differences in transcriptional expression between the two clones and during the AR development of each clone, so these formed six separate groups that were separated from each other.

### 2.4. Comparison of DEGs in Different Developmental Stages of T. hybrid ‘Zhongshanshan 149’ and T. hybrid ‘Zhongshanshan 118’

A total of 7720 DEGs were detected between 0 and 28 days *T. hybrid* ‘Zhongshanshan 149’ cuttings, with more upregulated than downregulated genes. From 28 to 56 days after cutting, 4286 DEGs were detected, most of which were downregulated. In total, 8245 DEGs were detected between samples 118–0 d and 118–28 d and 5959 DEGs between samples 118–0 d and 118–28 d, with both stages showing more upregulated than downregulated genes (Figures S2–S5). The number of DEGs was relatively high during callus formation but decreased in the rooting stage.

To further understand the functional classes and functional abundance of genes in the two stages of AR formation in *T. hybrid* ‘Zhongshanshan’, GO and KEGG enrichment analyses were carried out on the DEGs at two developmental stages of *T. hybrid* ‘Zhongshanshan 149’ and *T. hybrid* ‘Zhongshanshan 118’. In the GO analysis (Figures S6–S9), DEGs occurred during the callus formation (149–28 d/149–0 d) stage in *T. hybrid* ‘Zhongshanshan 149’, most of which were enriched in response to oxidative stress, copper ion binding, and peroxidase activity. In the rooting (149–56 d/149–28 d) stage of *T. hybrid* ‘Zhongshanshan 149’, DEGs occurred in the plant-type cell wall, cell wall organization, and copper ion binding categories. In the callus formation (118–28 d/118–0 d) stage of *T. hybrid* ‘Zhongshanshan 118’, DEGs were mainly enriched in the recognition of pollen, oxidoreductase activity, and response to salicylic acid categories. In the rooting (samples 118–56 d/118–28 d) stage of *T. hybrid* ‘Zhongshanshan 118’, DEGs occurred in the plant-type cell wall response to oxidative stress and protein kinase activity. By contrast, DEGs were mainly enriched in the plant-type cell wall, response to oxidative stress, and protein kinase activity categories.

KEGG enrichment analyses (Figures S10–S13) showed that DEGs were significantly enriched in the phenylpropanoid biosynthesis and flavonoid biosynthesis categories in the 149–28 d/149–0 d comparison. In the 149–56 d/149–28 d comparison, metabolic pathways were more enriched than in the 149–28 d/149–0 d comparison, and DEGs occurred in the starch and sucrose metabolism categories. In the 118–28 d/118–0 d comparison, DEGs were significantly enriched in phenylpropanoid biosynthesis, starch and sucrose metabolism, plant hormone signal transduction, and other metabolic pathways, whereas they were significantly enriched in the phenylpropanoid biosynthesis, biosynthesis of amino acids, starch, and sucrose metabolism pathways in the 118–56 d/118–28 d comparison.

### 2.5. Comparison of DEGs between Different Clones

**Figure 5:**
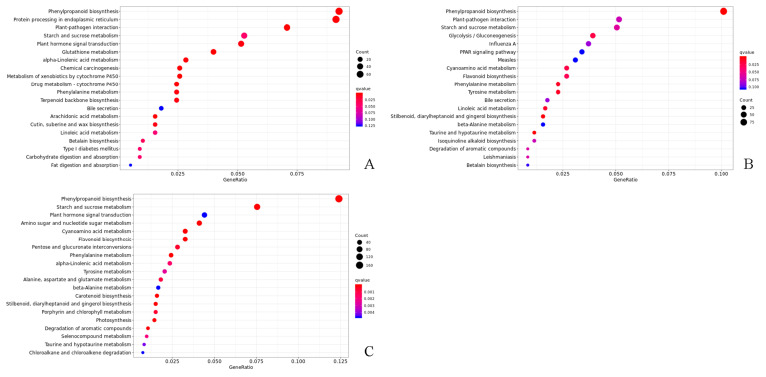
KEGG enrichment analysis of DEGs in the (**A**) 149–0 d/118–0 d, (**B**) 149–28 d/118–28 d, and (**C**) 149–56 d/118–56 d comparisons of *T. hybrid* ‘Zhongshanshan 149’ and *T. hybrid* ‘Zhongshanshan 118’.

Overall, 5033, 5060, and 7902 DEGs were, respectively, found at 0, 28, and 56 days after cutting across both forms of *T. hybrid* ‘Zhongshanshan’. The results indicated that there were differences in gene transcripts during rooting in *T. hybrid* ‘Zhongshanshan’. The DEGs recorded in each type of *T. hybrid* ‘Zhongshanshan’ and at different points after cutting establishment were subjected to KEGG enrichment analysis to reveal key genes involved in rooting. KEGG enrichment showed that DEGs in the 149–0 d/118–0 d, 149–28 d/118–28 d, and 149–56 d/118–56 d comparisons were mainly enriched in phenylpropanoid biosynthesis, with those in 149–56 d/118–56 d also enriched in starch and sucrose metabolism (Figure 5).

**Figure 6:**
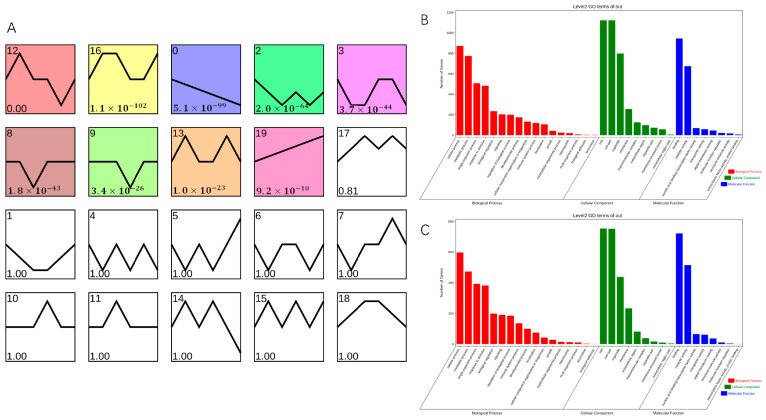
Transcript abundance of DEGs. (**A**) Clustering analysis of the DEGs into 20 clusters according to their expression profile. Comparison of GO enrichment of (**B**) cluster 0 and cluster 19 (**C**). Colored clusters were statistically significant (*p* < 0.05).

To determine genes related to AR formation, STEM software on OmicShare tools platform (https://www.omicshare.com/, accessed on 16 April 2023) was used to classify into 20 clusters according to their expression patterns; of these, nine clusters showed significant enrichment (Figure 6A). Cluster 0 comprised 2250 DEGs, which were downregulated during AR formation in both *T. hybrid* ‘Zhongshanshan 149’ and *T. hybrid* ‘Zhongshanshan 118’, although more so in *T. hybrid* ‘Zhongshanshan 118’ than in *T. hybrid* ‘Zhongshanshan 149’. Cluster 19 comprised 1906 DEGs, which were upregulated during AR formation in both types of *T. hybrid* ‘Zhongshanshan’, although more so in *T. hybrid* ‘Zhongshanshan 118’ than in *T. hybrid* ‘Zhongshanshan 149’.

GO enrichment of these two gene clusters was also performed (Figure 6B,C). The 2250 DEGs in cluster 0 were analyzed by GO enrichment (Figures S14–S16), and the top three genes were selected for comparative demonstration based on their gene number, p-value, and richness factor. It was found that 423, 37, and 483 DEGs related to stress, photosynthesis, and response to stimulus, respectively, were significantly enriched at the BP level. There were 67, 265, and 20 DEGs enriched at the CC level in the thylakoid, plastid, and nuclear envelope categories, respectively. At the MF level, DEGs were mainly concentrated in the transferase activity, ion binding, transferase activity, and transferring phosphorus-containing groups (342, 795, and 236 DEGs, respectively). GO analysis of the 1906 DEGs enriched in cluster 19 (Figures S17–S19) showed that 135, 336, and 382 DEGs were significantly enriched at the BP level in the immune system process, response to stress, and response to stimulus categories, compared with 233, 233, and 267 DEGs enriched at the CC level in the plasma membrane, membrane, and cell periphery categories, respectively, and 643, 166 and 202 DEGs at the MF level in the ion binding, kinase activity, transferase activity, and transferring phosphorus-containing group categories, respectively. Comparison of clusters 0 and 19 showed that significantly up- or downregulated genes were concentrated in response to stress, response to stimulus, ion binding, transferase activity, and transferring phosphorus-containing group categories.

### 2.6. Validation of Gene Expression Levels with qRT-PCR Analysis

**Figure 7:**
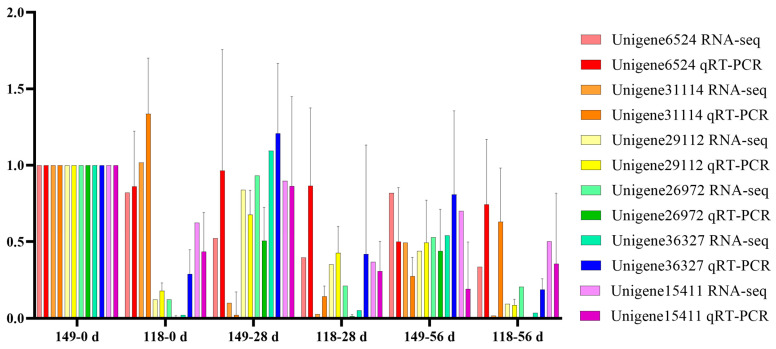
Verification of the gene expression quantity of randomly selected genes obtained from RNA-Seq using qRT-PCR.

To validate the transcript profiles produced in this study, 6 genes were randomly selected for qRT-PCR. The expression patterns of the 6 genes detected by qRT-PCR were consistent with those in the profiles of RNA-Seq, such as Unigene6524 was detected down and then upregulation pattern during AR formation of *T. hybrid* ‘Zhongshanshan 118’ both with qRT-PCR and RNA-Seq, which indicated the RNA-Seq data were reliable (Figure 7).

### 2.7. Cloning of the ThAP2 Gene

**Figure 8:**
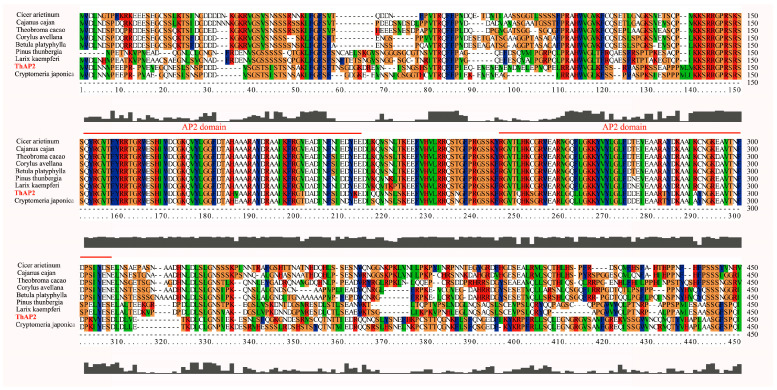
Alignment results of *ThAP2* with *AP2* protein of other species. The highly-conserved regions are represented by different colors and *AP2* domain is marked with red line.

Because the transcription factor *ThAP2* was found in cluster 19, which might be related to rooting, we further analyzed its function. We first designed primers based on the genome data and cloned them. The cloning results showed that the *ThAP2* gene contained 517 amino acids and had a very conserved *AP2* domain (Figure 8).

### 2.8. Subcellular Localization of ThAP2

**Figure 9:**
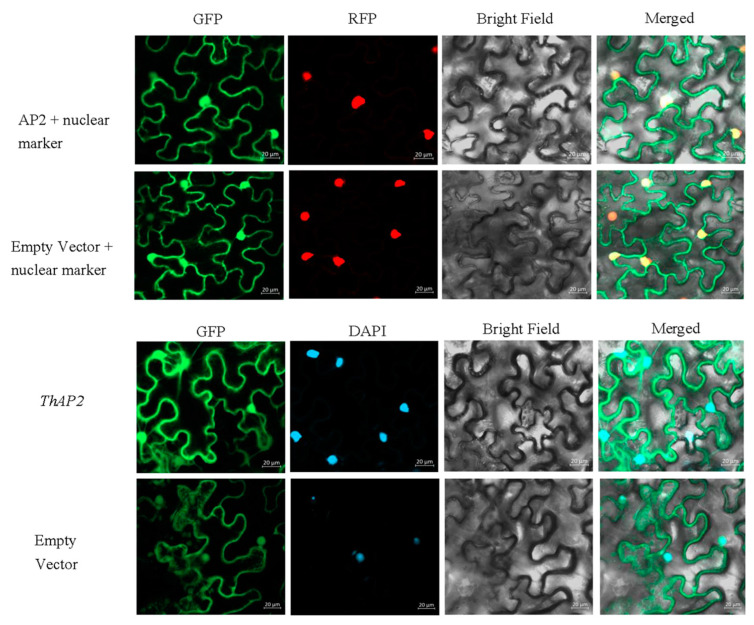
The result of subcellular localization of the *ThAP2* gene in the lower epidermis of *N. benthamiana* leaves.

Subcellular localization is a technique for studying where the gene functions. Referring to previous studies, subcellular localization of *ThAP2* was performed with tobacco leaf cells [9]. According to the results of laser confocal microscopy, *ThAP2* was localized in the nucleus (Figure 9); this location was further confirmed using nuclear markers and DAPI staining.

### 2.9. Gene Function Identification of ThAP2

**Figure 10:**
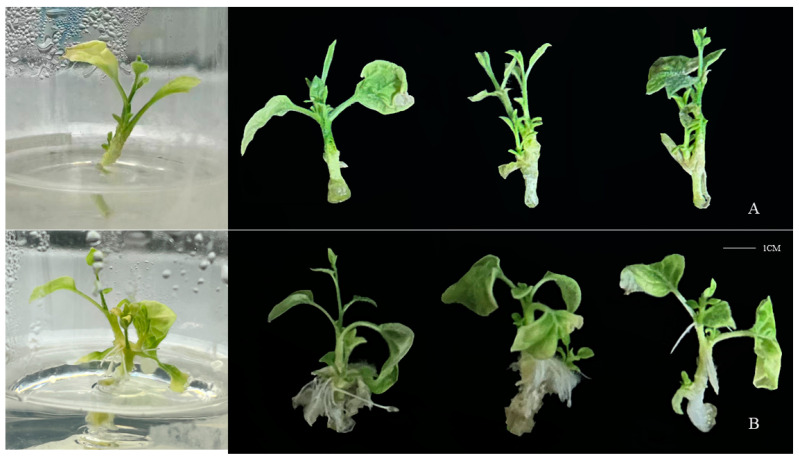
Phenotype of *ThAP2* in *N. benthamiana* after 20 days of culture. (**A**) wild-type *N. benthamiana* and (**B**) *ThAP2*-transgenic *N. benthamiana*.

The top buds of *ThAP2*-transgenic *N. benthamiana* were cut off for PCR and electrophoresis (Figure S20) and rooting culture, as well as the top buds from wild-type *N. benthamiana* as a control. After 20 days of culture, the transgenic plants had taken root, whereas the control group had not (Figure 10).

**Figure 11:**
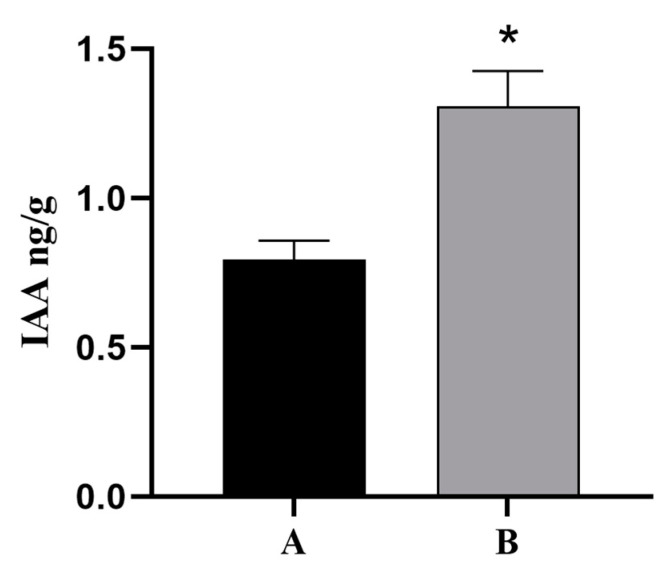
IAA Levels of *ThAP2* in base and root tissue of *N. benthamiana* cuttings. (**A**) wild-type *N. benthamiana* and (**B**) *ThAP2*-transgenic *N. benthamiana*, *p* < 0.05. “*” indicated a significant difference between the two sets of data.

Because plant hormone signal transduction pathways were involved in the regulation of cutting rooting in *T. hybrid* ‘Zhongshanshan’ and indoleacetic acid (IAA) is a vital growth hormone that can promote rooting, the base and root tissues of *ThAP2*-transgenic *N. benthamiana* cuttings were used as the material to determine the IAA levels, and the wild-type *N. benthamiana* was used as the control. The results showed that the content of IAA in the root tissue of transgenic plants was much higher than that in the control group (Figure 11).

## 3. Discussion

### 3.1. Quality Evaluation of Sampling and Sequencing

Successful propagation of cuttings depends on AR formation [8]. Previous observations revealed differences in the AR-forming ability of clones of *T. hybrid* ‘Zhongshanshan’, being poor in *T. hybrid* ‘Zhongshanshan 149’ but strong in *T. hybrid* ‘Zhongshanshan 118’ [10]. Such poor AR formation can limit the propagation of clones. In the current study, regular observations showed that numerous calli had formed 28 days after cutting, followed by numerous AR by Day 56 after cutting in *T. hybrid* ‘Zhongshanshan 118’, significantly more than in *T. hybrid* ‘Zhongshanshan 149’. Thus, these two time points (28 and 56 days post-cutting) were relevant to callus formation and AR formation, respectively. Such information was used for subsequent transcriptome analyses to explore AR formation in both kinds of *T. hybrid* ‘Zhongshanshan’ to identify DEGs involved in AR development.

The genetic samples obtained from tissues at the different time points were sequenced using Illumina HiSeq X ten, with 41,109 unigenes obtained through data filtering and quality assessment. Functional annotation of these unigenes showed that the highest hits in the Nr database were obtained with *Picea sitchensis* (Figure S1), which was consistent with the results of Wang et al. [10]. GO analysis showed that the unigenes were significantly annotated in response to stress, the nucleus, and ion binding. KEGG analysis showed that the DEGs were involved in signal transduction, translation, carbohydrate metabolism, and the endocrine system. Cutting mechanically destroys the plant stem, triggering the development of AR, which could be seen as a stress response [11], so many unigenes were annotated in response to stress items. A mount of unigenes is annotated in carbohydrate metabolism because carbohydrate metabolism provides essential substances and energy for rooting for the development of AR [12].

### 3.2. DEG Variation during AR Formation in the Two T. hybrid ‘Zhongshanshan’

Numerous DEGs were revealed in the two types of *T. hybrid* ‘Zhongshanshan’, with significantly more being found 56 days after cutting compared with 28 days and 0 days. There were significant differences between the two types of *T. hybrid* ‘Zhongshanshan’ at the transcriptome level. GO and KEGG enrichment analyses showed that copper ion binding, peroxidase, and oxidoreductase activity, response to oxidative stress, phenylpropanoid, and flavonoid biosynthesis, and some plant hormone signal transduction pathways, among others, were significantly different between the two types of *T. hybrid* ‘Zhongshanshan’. As indicated above, taking cuttings can be regarded as causing plant abiotic stress [11]. When the surface of cuttings is exposed to the surrounding environment, numerous reactive oxygen species (ROS) are produced, resulting in oxidative damage [13]. This activates peroxidase and oxidoreductase in response to oxidative stress [14,15]. As important secondary metabolites of plants, phenylpropanoid, and flavonoid act to resist strong oxidation activity and are often produced during stress stimulation and plant development [16]. Thus, in accordance with these observations, cuttings of both *T. hybrid* ‘Zhongshanshan’ showed resistance to ROS during their development. In addition, plant hormone signal transduction was enriched during the development of *T. hybrid* ‘Zhongshanshan 118’ but was absent in *T. hybrid* ‘Zhongshanshan 149’, which might explain why *T. hybrid* ‘Zhongshanshan 149’ did not take root. Plant hormones involved in rooting include IAA [17,18], gibberellic acid (GA) [19], and cytokinin (CTK) [18], ethene (ETH) [19]. Of these, the IAA directly regulates AR development [20].

STEM software was used to perform clustering analysis based on the expression patterns of genes to determine the genes involved in AR formation. Of the significant clusters, DEGs within clusters 0 and 19 showed the same expression trends, being down- and upregulated, respectively. The significantly up- or downregulated genes focused on response to stress, response to stimulus, ion binding, transferase activity, transferring phosphorus-containing groups, and transcription factors, such as *AP2*. The mechanical damage caused by taking cuttings is one reason for the frequent enrichment of genes related to stress and the stress and stimulus-response [11]. Genes involved in transferring phosphorus-containing groups were continuously upregulated, indicating that oxidative phosphorylation occurs during cutting and that a significant amount of transferase is needed to regulate it. By comparison, the genes involved in phosphotransferase activity were more enriched in *T. hybrid* ‘Zhongshanshan 118’ than in *T. hybrid* ‘Zhongshanshan 149’, which might also be a reason for the difference in AR formation. This was consistent with other woody plants. For example, in *Eucalyptus urophylla*, genes related to oxidative phosphorylation were significantly enriched in a slow-rooting group among clones with different rooting abilities [21].

### 3.3. Gene Function Identification of ThAP2

In this study, one upregulated gene (Unigene 17,167: *ThAP2*), known to be involved in rooting, was found in cluster 19, indicating that it might be involved in AR formation. The *AP2/ERF* family is one of the largest plant transcription factors, and its coding genes, such as *PLT*, *AIL*, and *BBM*, have been reported to be involved in rooting [22,23,24]. In total, 157 *AP2/ERF* genes were identified in oily persimmon (*Diospyros oleifera*) from four subfamilies (*AP2*, *RAV*, *Soloist,* and *ERF*), among which *DkAP2/ERF* was expressed most in the root [25]. Genes such as *OsAP2/ERF-40* might also determine the fate of root cells because these genes are specifically expressed in the AR primordium [26]. Therefore, this study focused on the cloning, subcellular localization, and function of *ThAP2*. We found that DEGs were significantly enriched in plant hormone signal transduction. Studies have shown that higher levels of IAA can promote AR growth during the development of AR [27]. IAA could affect AR development by acting directly on cell components [20], while other hormones regulate AR development by interacting with IAA to induce primordial roots [27]. In plants such as tea [17], IAA has a great relationship with rooting and promotes the elongation of AR. Therefore, the determination of IAA levels was one of the important indexes for the function of *ThAP2* in this study.

This gene was transferred into *N. benthamiana* and its subcellular localization, the resulting rooting phenotype, and IAA levels were determined. The results of subcellular localization showed that *ThAP2* was localized to the nucleus, whereas phenotype observation results showed that the transgenic plants were easy to root and found that the IAA level of transgenic plants was higher than wild-type, indicating that *ThAP2* has a positive effect on AR formation. The current results further our understanding of the key regulatory factors involved in AR formation and can provide candidate gene resources for developing genetic breeding strategies for optimizing superior clones of *T. hybrid* ‘Zhongshanshan’ [28].

## 4. Materials and Methods

### 4.1. Plant Materials

Softwood cuttings of *T. hybrid* ‘Zhongshanshan 149’ and *T. hybrid* ‘Zhongshanshan 118’ were collected from the cutting orchard at the National Improved Seed Breeding Base of *T. hybrid* ‘Zhongshanshan’, Jingjiang Jiangsu, China. Healthy softwood cuttings with similar growth potential were trimmed to a length of 15–18 cm; before cutting, half of the leaves were removed from each cutting to prevent excess water loss. The selected seedbed was 90 cm wide × 30 cm high, and the culture medium was peat soil with organic matter and moistened perlite in a 1:1 ratio. To prevent mold and other fungal attacks, the culture medium was thoroughly sprayed with 1000 mg/L carbendazim before cutting. On 5 June 2020, 100–120 softwood cuttings were planted neatly into the seedbed. The cuttings were observed every 7 days to detect the two key stages of rooting: callus formation stage and rooting stage. The callus formation rate is the percentage of cuttings with callus formation in total cuttings. The rooting rate is the percentage of cuttings with AR formation in total cuttings.

Sampling was performed in accordance with the test results in 2.1. At each time point, 0.5 g of stem base cortex and root tissue was removed from each cutting with a knife, immediately frozen with liquid nitrogen, and stored at −80 °C for later use. Three samples were taken for each time point.

### 4.2. Total RNA Isolation and Sequencing

The total RNA of each sample was extracted with TRIzol reagent (Invitrogen, Carlsbad, CA, USA). The purity and integrity of RNA were monitored using agarose gels, and the concentration and quality of RNA were accurately detected using the bioanalyzer system (Agilent, 2100, Santa Clara, CA, USA). The sequencing library was constructed by NBE. Using total RNA, mRNA with polyA tails was enriched with Oligo (dT) magnetic beads and then randomly interrupted by divalent cations in the Fragmentation Buffer. The first-strand cDNA was then synthesized in the M-MuLV reverse transcriptase (Vazyme, Nanjing, China) system using the mRNA fragments as templates and random oligonucleotides as primers. The RNA was then decomposed by RNaseH, and the double-stranded cDNA was synthesized using dNTPs as raw material. The RNA was then decomposed by RNaseH, and the double-stranded cDNA was synthesized using dNTPs as raw material in the DNA polymerase system (Vazyme, Nanjing, China). Finally, AMPure XP beads (Vazyme, Nanjing, China) were used to bind cDNA fragments of ~370–420 base pairs for PCR amplification and purification to obtain a sequencing library. After the library was constructed, a Qubit2.0 Fluorometer (Colibri LB 915, Berlin, Germany) was used for initial quantification, and the library was diluted to 1.5 ng/μL. The insert size of the library was then tested using an Agilent 2100 bioanalyzer. Quantitative real-time PCR (qRT-PCR) was used to accurately quantify the effective concentration of the library (which was >2 nM) to ensure its quality.

The cDNA library was sequenced using Illumina HiSeq X ten (Beina Gene, Wuhan, China) based on double-ended sequencing using the basic principle of sequencing by synthesis. All low-quality adapter/primer contaminants and duplicated reads were removed. The sequencing error rate and GC content distribution were checked, resulting in high-quality clean reads, which were then spliced using the Trinity software (version: 2.6.6) package and their transcripts obtained. N50, ExN50, and BUSCO (version: 1.22) were used to evaluate the assembly results to ensure their accuracy and effectiveness. In subsequent experimental analyses, the longest transcript of each gene was selected as the unigene.

### 4.3. Bioinformatic Analysis of RNA-Seq Data

Functional annotation of the unigenes was performed to obtain comprehensive gene function information. BLAST (version: 2.2.31+) was used to compare the relationship between all protein-coding genes and the UniProt, KEGG, GO, Nr, Pfam, and EggNOG databases. UniProt and Nr annotations were made using diamond (version: 0.9.24; parameter: max-target-seqs 1, e-value 1 × 10^−5^) BLASTx to match unigenes to those in the database; the unigenes were then annotated according to the comparison result. GO, KEGG, and EggNOG unigene annotations were all based on the association between the Uniprot and Swiss-Port databases. Pfam annotations were made using hmmscan (version: 3.1; parameter: e-value 0.01) to predict conservative domains.

Transcriptome data were compared to identify genes with different expression levels in different samples. Clean reads were mapped to unigenes using the Bowtie2 software package (version: 2.3.4; parameter: mismatch 0). Then, RSEM (version: 1.3.1) was used to estimate the gene expression levels for each sample. A principal component analysis (PCA) was performed on the obtained data, using 149–0 d/118–0 d, 149–28 d/118–28 d, 149–56 d/118–56 d, 149–28 d/149–0 d, 149–56 d/149–28 d, 118–28 d/118–0 d, and 118–56 d/118–28 d as the comparison groups. The differential expression analysis software used was DESeq2 (version: 1.26.0), screening threshold for q-value < 0.05 and |log_2_FoldChange| > 1. Additionally, clusterProfiler (version: 3.14.3) was used to analyze the enrichment of the DEGs and to determine the GO term or KEGG pathway of each DEG compared with all enrichment genes. Short Time-series Expression Miner software (STEM) on the Omic Share tools platform (https://www.omicshare.com/, accessed on 16 April 2023) was also used to analyze the gene expression patterns. The functional analysis of the DEGs was performed using GO analysis. Heat maps representing the gene expression levels were created using TBtools (version: 1.045).

### 4.4. qRT-PCR Validation of the RNA-Seq Results

Six functional genes were selected at random using qRT-PCR to verify the results of RNA-Seq (Table S3). The experimental materials and RNA preparation methods were the same as those used for the RNA-Seq described earlier. A SuperScript II reverse transcriptase kit (TaKaRa, Dalian, China) was used to synthesize cDNA. qRT-PCR was performed with a StepOnePlus Real-Time PCR system (2012 Life Technologies Corporation, Carlsbad, CA, USA, version 2.3). The RT-PCR program was as follows: 30 s at 95 °C; 40 cycles of 95 °C for 10 s, 60 °C for 30 s; 95 °C for 15 s–60 °C for 60 s–95 °C for 15 s was used to generate a melting curve. The reaction mixture in a 20-μL volume contained the following: 10 μL of 2 × ChamQ Universal SYBR qPCR Master Mix (Vazyme, Nanjing, China), 1 μL of template cDNA, 0.4 μL of each primer, and 8.2 μL ddH_2_O. Three replicate analyses were performed for each reaction, and then the data were analyzed using the 2^−ΔΔCT^ method [29]. The transcript level of ThAPRT served as the internal standard (Table S3).

### 4.5. Gene Cloning and Subcellular Localization

The sequence of *ThAP2* gene, which might be related to rooting [25,26], was obtained from the transcriptomic data, and primers were designed using Oligo 6 software according to the sequence (Table S4). Primer synthesis was completed by General Biological (Anhui, China). La Taq (Takara, Dalian, China) was used to amplify the full-length sequences, and pMD19-T vectors (Takara, Dalian, China) were used for TA cloning. ClustalW (version: 2.1) software was used to perform protein sequence comparisons. Using a ClonExpress II One Step Cloning Kit (Vazyme, Nanjing, China), *ThAP2* full-length sequences were inserted into the pEarleyGate 101 vector to form a recombinant vector and then transferred into *Agrobacterium* EHA105 by electric conversion. A single *Agrobacterium* colony was selected to prepare a heavy suspension with OD 600 = 0.6. This was then injected into the lower epidermis of *N. benthamiana* leaves, which had been seeded in advance, with a 1-mL syringe without a gun head. After 2 days of low-light culture, slides were prepared of the lower epidermis with tweezers, which were then observed and photographed under an Olympus LEXT laser confocal microscope.

### 4.6. Phenotypic Verification and IAA Levels of ThAP2 in N. benthamiana

The genetic transformation system of *T. hybrid* ‘Zhongshanshan’ has not yet been established. Therefore, *ThAP2* transgenic seedlings of *N. benthamiana* were used to verify the gene function in this study. *ThAP2* plasmids were transferred into *Agrobacterium* GV3101-receptive cells by electro-transformation, and the cells were then cultured with LB medium. *N. benthamiana* seeds were disinfected with 75% alcohol, 84% disinfectant, and sterile water, sown on the germination medium, and then cultured. After the seedlings had grown to 7–10 cm, the leaves were cut into small pieces with a scalpel and inoculated on pre-medium. A single Agrobacterium was selected in the infection solution, and a heavy suspension of *Agrobacterium* with OD 600 = 0.2 was prepared. Transgenic plants were obtained by infection, co-culture, callus induction, and two antibiotic resistance screening and seedling-strengthening operations. The top buds of wild-type and transgenic plants were cut and placed in a rooting medium. The growth of AR in the medium was observed every 3 days to determine the function of *ThAP2* in AR formation in *T. hybrid* ‘Zhongshanshan’. The residual culture medium on the plant surface was rinsed off, and 2–3 cm sections of root tissue were collected at a weight of 0.5 g per sample. This sampling process was repeated 3 times, immediately frozen with liquid nitrogen, and stored at −80 °C for later use. The endogenous phytohormones of samples were extracted by isopropanol, H_2_O, and HCL, and the contents of IAA were determined by high-performance liquid chromatography (HPLC, Agilent 1290, https://www.agilent.com/, accessed on 4 December 2023) and tandem mass spectrometry (MS/MS, Applied Biosystems 6500 Quadrupole Trap, https://sciex.com.cn/, accessed on 4 December 2023). The internal standard substance was added to the extraction solution to correct the detection result.

### 4.7. Statistical Analysis

All experiments included three biological duplications and Student’s *t*-test was used for significant difference analysis, * *p* < 0.05.

## 5. Conclusions

Using the Illumina sequencing platform, transcriptome sequencing was performed on base and root tissues of *T. hybrid* ‘Zhongshanshan 149’ and *T. hybrid* ‘Zhongshanshan 118’ cuttings. Significant gene programming differences in the formation of AR were recorded between the two types of *T. hybrid* ‘Zhongshanshan’. *ThAP2* was identified as a conserved gene that appears to be regulated during AR formation at different time points in each clone of *T. hybrid* ‘Zhongshanshan’. Transgenic seedlings of *N. benthamiana* were used to verify the function of *ThAP2*. These results provided a reference for the development of AR formation in woody plants and could be used to develop genetic breeding strategies to optimize clones of *T. hybrid* ‘Zhongshanshan’.

## Figures and Tables

**Table 1:** Callus formation and rooting rate of *T. hybrid* ‘Zhongshanshan 118’ and of *T. hybrid* ‘Zhongshanshan 149’ at different time points after cutting establishment.

Sample Name	Primitive Cutting Period Rate	Callus Formation Rate	Rooting Rate
149–0 d	100.00%	–	–
118–0 d	100.00%	–	–
149–28 d	94.00%	6.00%	–
118–28 d	15.58%	84.42%	–
149–56 d	67.50%	30.83%	1.67%
118–56 d	0.01%	37.60%	62.39%

**Table 2:** Statistical characteristics of the resulting unigenes.

Characteristic	Value
Total_length (bp)	58,678,213
Total_number	41,109
GC_content (%)	40
N50 (bp)	2343
N90 (bp)	641
Average (bp)	1427.38
Median (bp)	935
Min (bp)	201
Max (bp)	17,892

Total_length is the total length of the assembly sequence; Total_number is the total number of assembly sequences; GC_content is the average GC content of the assembly sequence; N50 is the sequence arranged in descending order of length and accumulated sum. The length of the corresponding sequence is calculated when the length reaches 50% of the total length; N90 is the sequence arranged in descending order of length and accumulated sum. The length of the corresponding sequence is calculated when the length reaches 90% of the total length; “average” is the average length of the assembly sequence; “Median”, “Min”, and “Max” indicate the median, minimum, and maximum lengths, respectively, of the assembly sequence.

## Data Availability

The data have been deposited to the National Center for Biotechnology Information (NCBI) under accession numbers PRJNA1057719 and PRJNA1057810.

## Supplementary Materials

The following supporting information can be downloaded at: https://www.mdpi.com/article/10.3390/ijms25042427/s1.
