# Supplementary material for: Molecular Mechanism of Different Rooting Capacity between Two Clones of Taxodium hybrid ‘Zhongshanshan’
Source: Int J Mol Sci. 2024 Feb 19;25(4):2427. doi: 10.3390/ijms25042427 (PMC10889566; doi:10.3390/ijms25042427)
Supplement: Supplementary file 1 [file ijms-25-02427-s001.zip › Supplementary Figures/FigureS1 to S20.pdf/Figure S1. Nr annotation of the combined transcriptome data from T. hybrid 'Zhongshanshan 149' and T. hybrid 'Zhongshanshan 118'.pdf]

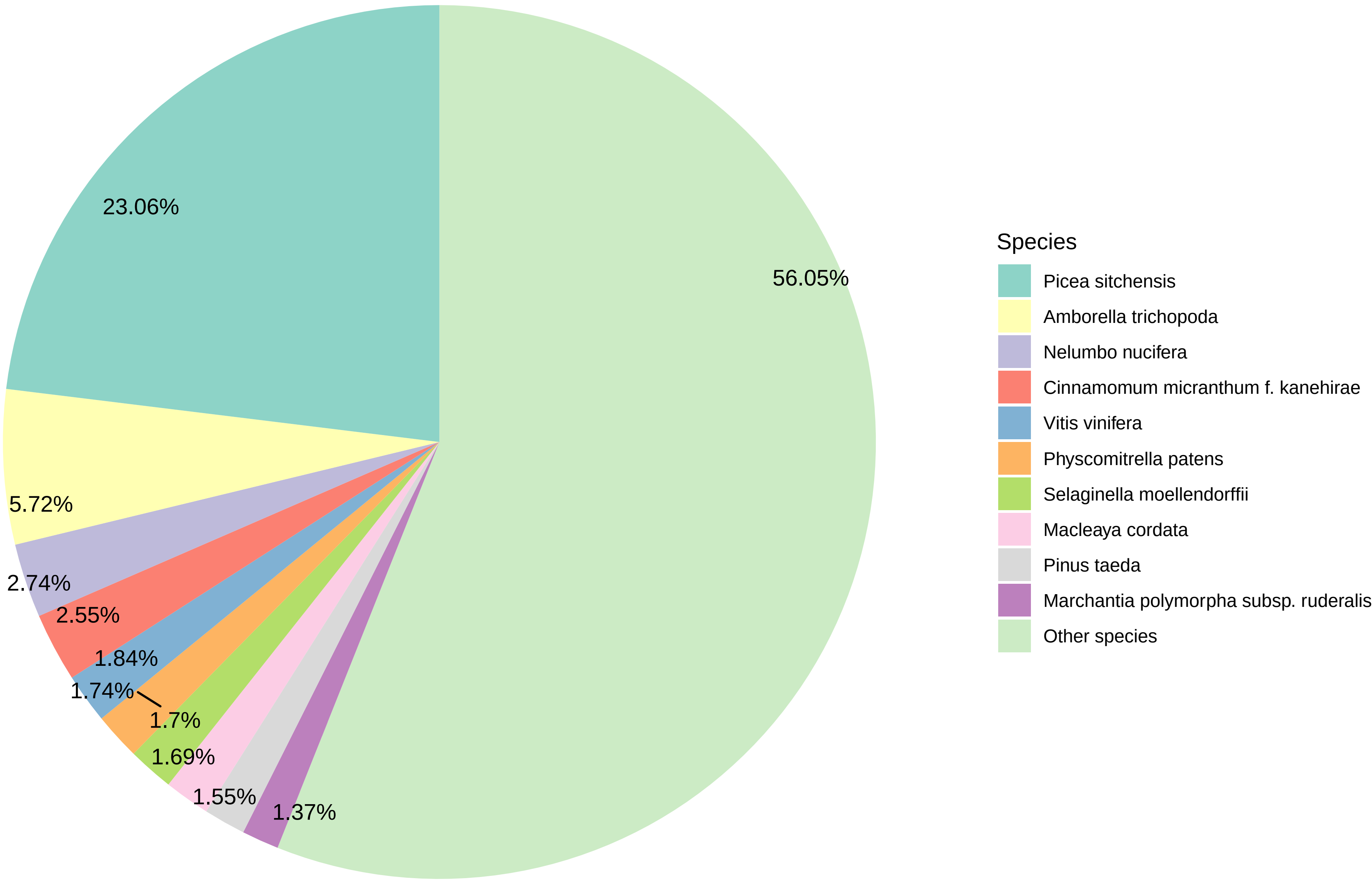


**Figure S1.** Nr annotation of the combined transcriptome data from *T. hybrid* 'Zhongshanshan 149' and *T. hybrid* 'Zhongshanshan 118'.
