# Supplementary material for: Molecular Mechanism of Different Rooting Capacity between Two Clones of Taxodium hybrid ‘Zhongshanshan’
Source: Int J Mol Sci. 2024 Feb 19;25(4):2427. doi: 10.3390/ijms25042427 (PMC10889566; doi:10.3390/ijms25042427)
Supplement: Supplementary file 1 [file ijms-25-02427-s001.zip › Table S1. Summary of the clean reads data.pdf]

Table S1. Summary of the clean reads data

Sample_name	Total_reads	Total_bases	GC_content	Q20	Q30
149-0 d-1	44,730,322	6,709,548,300	43.50%	98.06%	94.16%
149-0 d-2	40,402,440	6,060,366,000	43.36%	97.39%	92.64%
149-0 d-3	42,377,504	6,356,625,600	43.29%	97.82%	93.58%
118-0 d-1	42,238,650	6,335,797,500	43.12%	97.49%	92.80%
118-0 d-2	38,289,070	5,743,360,500	43.31%	97.49%	92.83%
118-0 d-3	45,227,224	6,784,083,600	43.30%	97.74%	93.47%
149-28 d-1	40,920,626	6,138,093,900	44.09%	97.36%	92.64%
149-28 d-2	40,673,634	6,101,045,100	49.86%	97.10%	92.37%
149-28 d-3	42,557,856	6,383,678,400	43.48%	97.50%	92.87%
118-28 d-1	39,647,060	5,947,059,000	43.51%	97.43%	92.76%
118-28 d-2	46,371,278	6,955,691,700	43.63%	97.63%	93.22%
118-28 d-3	43,072,894	6,460,934,100	43.56%	97.71%	93.37%
149-56 d-1	39,086,508	5,862,976,200	43.16%	97.30%	92.47%
149-56 d-2	45,272,780	6,790,917,000	43.29%	97.67%	93.33%
149-56 d-3	40,304,572	6,045,685,800	43.45%	97.49%	92.87%
118-56 d-1	45,976,824	6,896,523,600	43.18%	97.70%	93.38%
118-56 d-2	44,998,956	6,749,843,400	43.53%	97.70%	93.38%
118-56 d-3	42,407,586	6,361,137,900	45.16%	97.24%	92.39%

Note: Total_Reads is the number of reads (a pair of paired-end reads is denoted as two reads); Total_bases is the total base number; GC_content is the percentage of the number of bases G and C in the total number of bases; Q20 and Q30 are the percentage of bases with Phred values >20 and >30 in the total number of bases, respectively.
