# Supplementary material for: Molecular Mechanism of Different Rooting Capacity between Two Clones of Taxodium hybrid ‘Zhongshanshan’
Source: Int J Mol Sci. 2024 Feb 19;25(4):2427. doi: 10.3390/ijms25042427 (PMC10889566; doi:10.3390/ijms25042427)
Supplement: Supplementary file 1 [file ijms-25-02427-s001.zip › Table S2. Summary of the functional annotations of the unigenes.pdf]

Table S2. Summary of the functional annotations of the unigenes

Description	No. of unigenes identified	Percentage identified
Total identified	41,109	100%
Total annotated	41,101	99.98%
**Database**		
the UniProt Knowledgebase (UniProt)	28,872	70.23%
Pfam	29,541	71.86%
Gene Ontology (GO)	28,506	69.34%
Kyoto Encyclopedia of Genes and Genomes (KEGG)	12,515	30.44%
KEGG Pathway	8,303	20.20%
EggNOG	23,586	57.37%
NCBI non-redundant protein sequences (Nr)	41,087	99.95%
