# Supplementary material for: Molecular Mechanism of Different Rooting Capacity between Two Clones of Taxodium hybrid ‘Zhongshanshan’
Source: Int J Mol Sci. 2024 Feb 19;25(4):2427. doi: 10.3390/ijms25042427 (PMC10889566; doi:10.3390/ijms25042427)
Supplement: Supplementary file 1 [file ijms-25-02427-s001.zip › Table S3. Primers used in qRT-PCR.pdf]

Table S3: Primers used in qRT-PCR

**Unigenes**	**Primer F**	**Primer R**
Unigene6524	TCTCAACCTTGGGCTTGGAT	GCAGTGCAAGTAAAGCTCCA
Unigene31114	TACTCTGCCCACGAACAACT	CAAAGAGCAAGCCAGCTGAT
Unigene29112	TGTCAAGGATTACCTTTGTCCCT	CTTGCAAGAGTGATTCCCAA
Unigene26972	GAGGAGCTGCGCTTAATGTT	TGTTTCCACGTAGCGGAGAT
Unigene36327	GCTTGGCATGAAATGGTTGC	AAGGACGGACTGGGATCAAA
Unigene15411	AGATGTGGCATGATGCTTGG	GCTTTCTGTGAGGGTTGCTT
ThAPRT	TCCACAGGTTCTTGAATCGCT	TGACTTGAGCCTCATTCGCTC
