# Supplementary material for: Molecular Mechanism of Different Rooting Capacity between Two Clones of Taxodium hybrid ‘Zhongshanshan’
Source: Int J Mol Sci. 2024 Feb 19;25(4):2427. doi: 10.3390/ijms25042427 (PMC10889566; doi:10.3390/ijms25042427)
Supplement: Supplementary file 1 [file ijms-25-02427-s001.zip › Table S4. Sequence and primers of ThAP2.pdf]

**Table S4:** Sequence and primers of *ThAP2*

| Sequence | Primer |
| --- | --- |
| ATGTGGGATCTGAACAATATGCCCGAGGAGCCTAGGCCTGTG | |
| GAAGTGGAGGGCCAAAACGAGAGCTTATCAAATTCGCCTGA | |
| TGATGACGTCTCGGGTTCGACTTCCATCTCTACCAGCAACTC | |
| TGCTAAGATCTTTGGGTTTTCTTTTACTAACAGTGGTGATGGA | |
| AAAGATCGGGAGATGAATATAAGTAATGGATCCCACAGCGTT | |
| ACACGCCAGTTTTTCCCAATTGAGCAGGAGATGGAGATGGA | |
| GATGGAGATGGATATGGAATTAGAGCCAGTACAGCCTGAAC | |
| TGCGAAGAGCCCATTGGGTAGGACTTAAGTTTTCTTCTCGGT | |
| GGGCTTCACCCAAATCCAGTGAAGCCCCTCCACCCATGCTCA | |
| TGAAGAAGAGTAGACGAGGCCCACGTTCCAGAAGTTCTCAG | |
| TACCGTGGTGTTACATTCTACAGGCGCACTGGAAGATGGGAG | |
| TCTCACATATGGGATTGTGGGAAACAAGTCTATCTTGGTGGA | |
| TTTGACACTGCCCACGTAGCCGCCAGGGCATATGATAGGGCA | |
| GCCATCAAATTCAGAGGCACGGATGCTGACATAAATTTTAGT | |
| ATTGATGACTATAAGGAGGACATCCAGCAGATGAATAGTTTG | |
| TCAAAGGAGGAGTTTGTGCATGTCCTTCGCCGGCAAAGCAA | Primer F : |
| TGGATTTCCAAGAGGAAGTTCTAAGTTCAGAGGCGTCACTC | ATGTGGGATCT |
| AGCACAAATCTGGAAGATGGGAAGCTAGACTTGGACAACTT | GAACAATATG |
| CTGGGCAAAAAGTATGTTTACTTAGGTTTATTCGACGACGAA | |
| ATCGAAGCTGCAAGGACATATGATAAAGCAGCAATTGCATA | Primer R: |
| CAACGGCAAGGAAGCGGTTACTAATTTTGATCCTAAAGTATA | AGAACCTAAGG |
| CGAAAGTGATCTAGATCTTGTAGAAACAAAAGATCTTTGTCT | ACAAGTTTAAT |
| TGGCAACTCTATTGAAAAAGAAAGCAACTTGTCATTTCAAG | AA |
| GCAAGGGAAACGATGAGAGCAGAATGCAGTCATGTCAAACT | |
| AATACCACGATTGAAGATAGGCAGCAAAATCAGAGCTTGTA | |
| TTCAAACGAGTTTCACAAGCCTTGTTCAACAACACAAGGCA | |
| ATAAGTTTCTGTCCTTCCAAAATGGGGAGGATTTTATCAAGTA | |
| CAAGAGGCCATTTAGGTTGTTATCCCAGTTGGAAGGTAATGG | |
| ACAATGGCTGGCAAGTGATGATGTGATTGTGCACCGAGGATC | |
| ATCAGGGAATCCACTGCCTTCTCTTTCTCACCTTAACTACAAG | |
| GGTCCAAGTATCTTGGGTGGATGCAGAGATGAGAGAAAAAG | |
| AGGAGTATCAGCGATGTTTGGAAGGGAGAAAGTATCATCAG | |
| GATGGGTTAATTGTCAAATGCAGACATATGTTCATGCTCCTCT | |
| CGCAGCATCATCAGGATTCTCACCCCAAATTCTATTCGCAAC | |
| AAATTCTTCTTCTGATTGCCAACAGAAAACAGGATTTCATGTT | |
| AAGATTCAACCTACATTGTATTCAAACAATTTGCATAAAACT | |
| ACTTCTTCAAAATCTGGTGACCACTTATTAAACTTGTCCTTAG | |
| GTTCTTAA | |
