# Supplementary figures and images for: Molecular Mechanism of Different Rooting Capacity between Two Clones of Taxodium hybrid ‘Zhongshanshan’
Source: Int J Mol Sci. 2024 Feb 19;25(4):2427. doi: 10.3390/ijms25042427 (PMC10889566; doi:10.3390/ijms25042427)

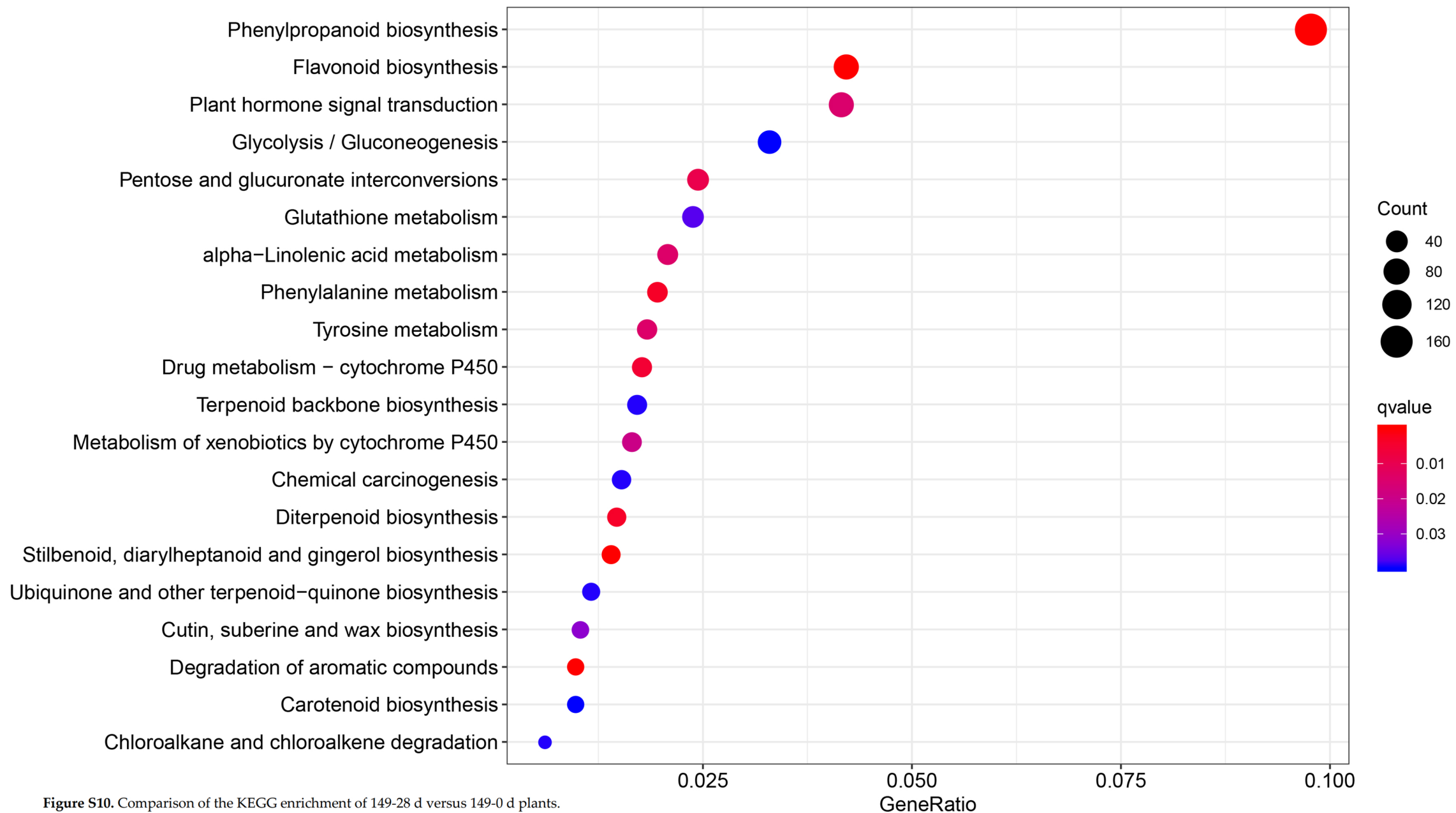

Supplement: Supplementary file 1 [file ijms-25-02427-s001.zip › Supplementary Figures/FigureS1 to S20.pdf/Figure S10. Comparison of the KEGG enrichment of 149-28 d versus 149-0 d plants.pdf]

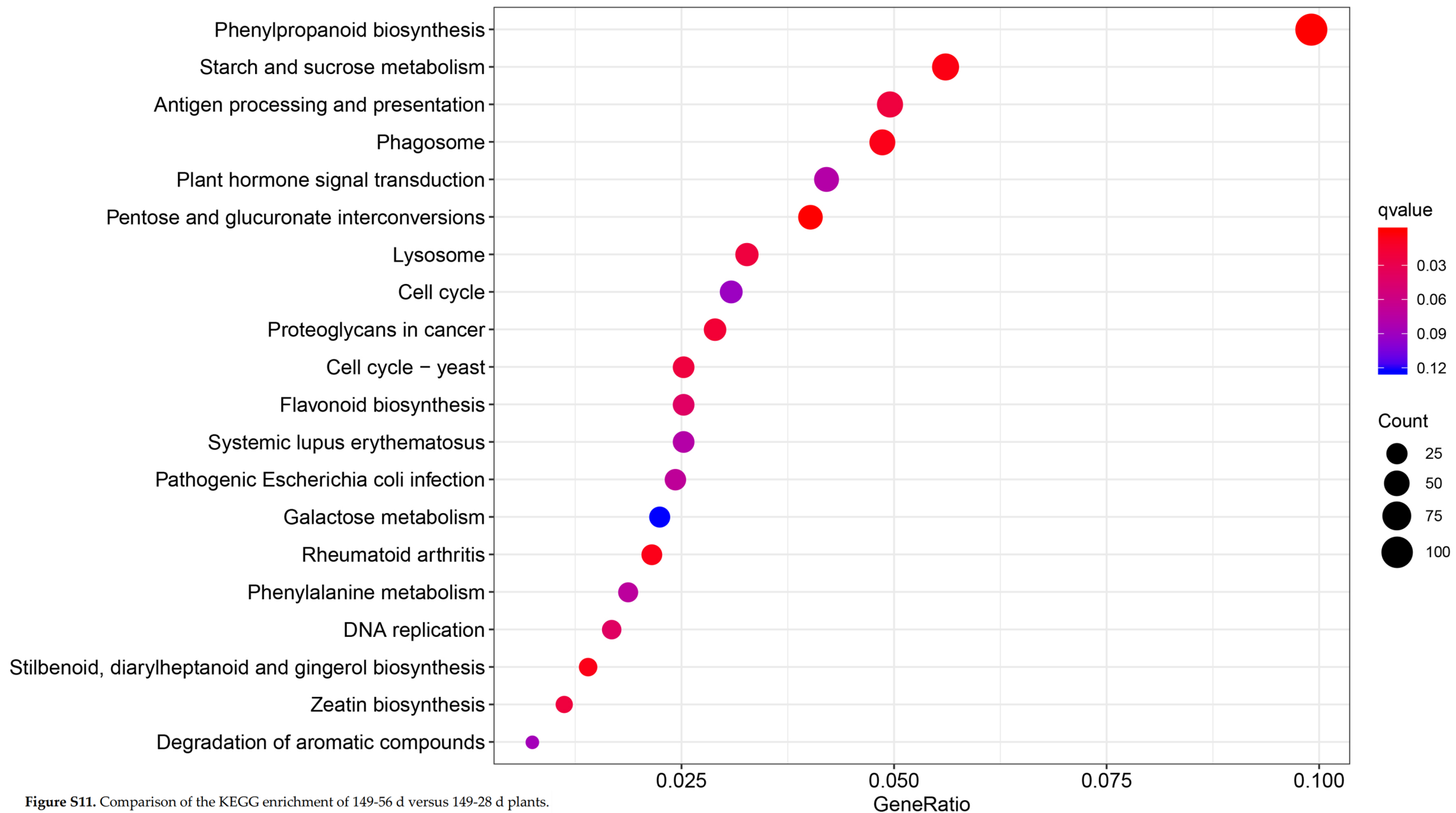

Figure S11. Comparison of the KEGG enrichment of 149-56 d versus 149-28 d plants.

Supplement: Supplementary file 1 [file ijms-25-02427-s001.zip › Supplementary Figures/FigureS1 to S20.pdf/Figure S11. Comparison of the KEGG enrichment of 149-56 d versus 149-28 d plants.pdf]

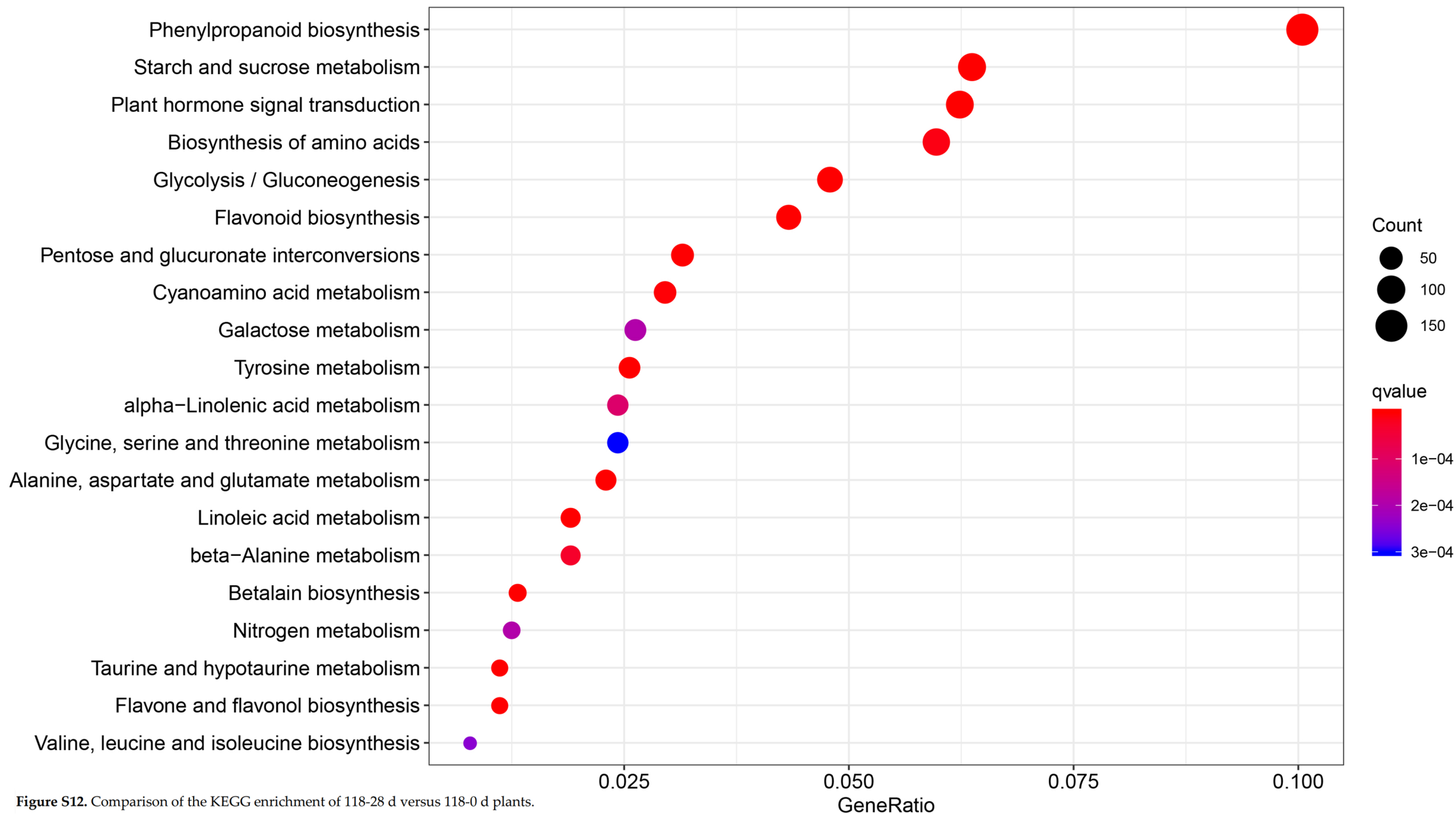

Supplement: Supplementary file 1 [file ijms-25-02427-s001.zip › Supplementary Figures/FigureS1 to S20.pdf/Figure S12. Comparison of the KEGG enrichment of 118-28 d versus 118-0 d plants.pdf]

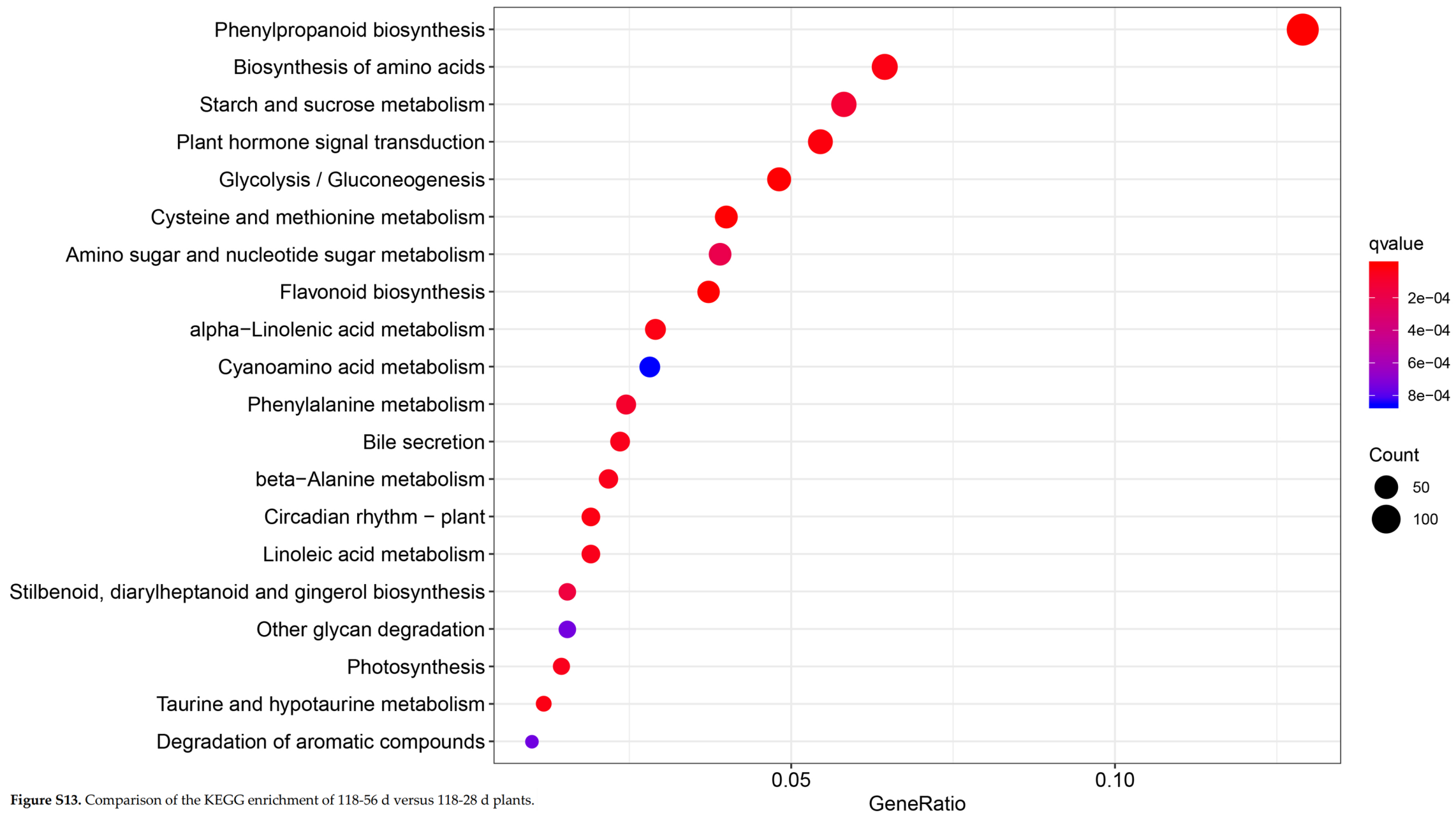

Supplement: Supplementary file 1 [file ijms-25-02427-s001.zip › Supplementary Figures/FigureS1 to S20.pdf/Figure S13. Comparison of the KEGG enrichment of 118-56 d versus 118-28 d plants.pdf]

# Top 25 of GO Enrichment

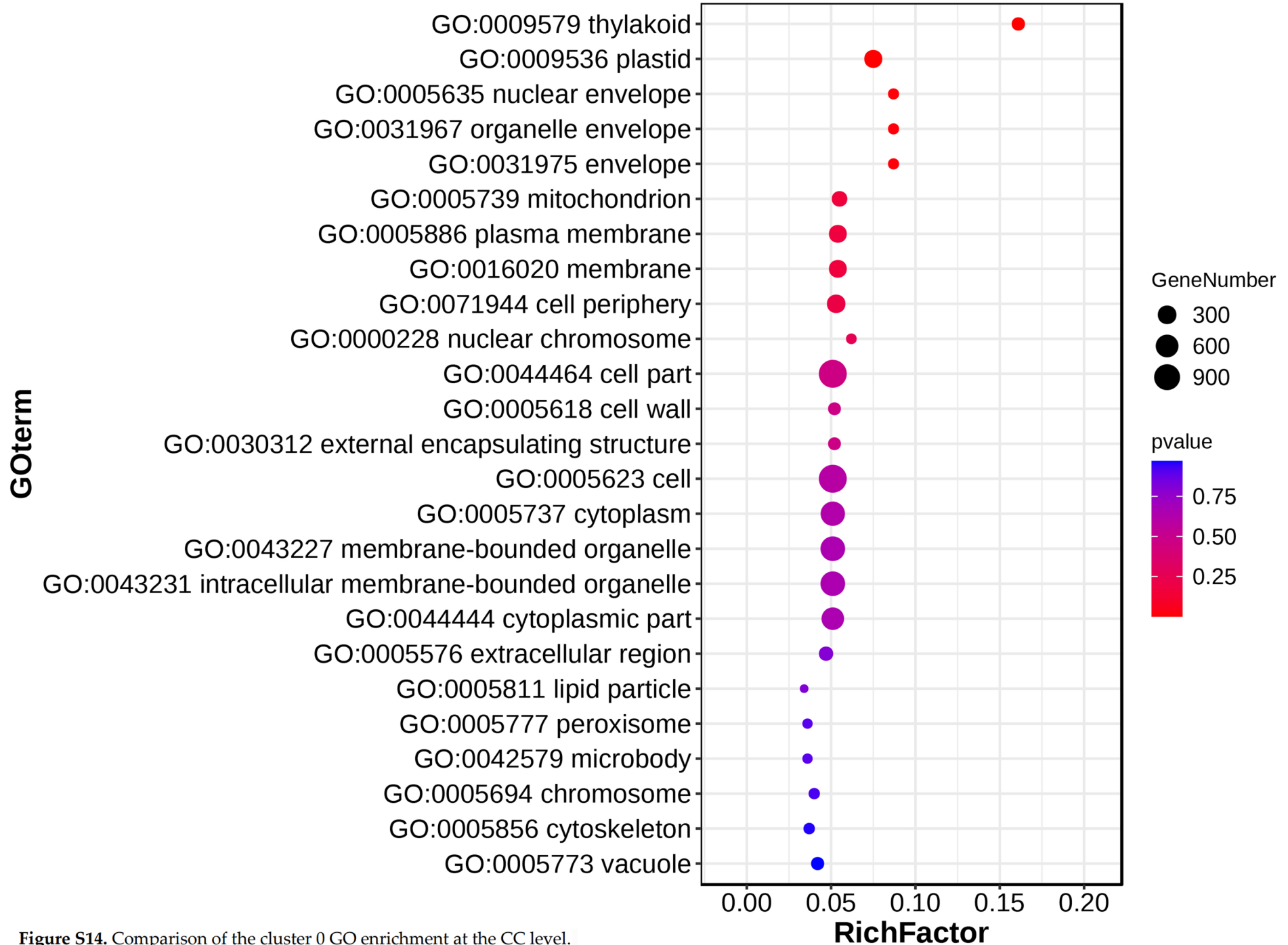

**Figure S14.** Comparison of the cluster 0 GO enrichment at the CC level.

Supplement: Supplementary file 1 [file ijms-25-02427-s001.zip › Supplementary Figures/FigureS1 to S20.pdf/Figure S14. Comparison of the cluster 0 GO enrichment at the CC level.pdf]

# Top 25 of GO Enrichment

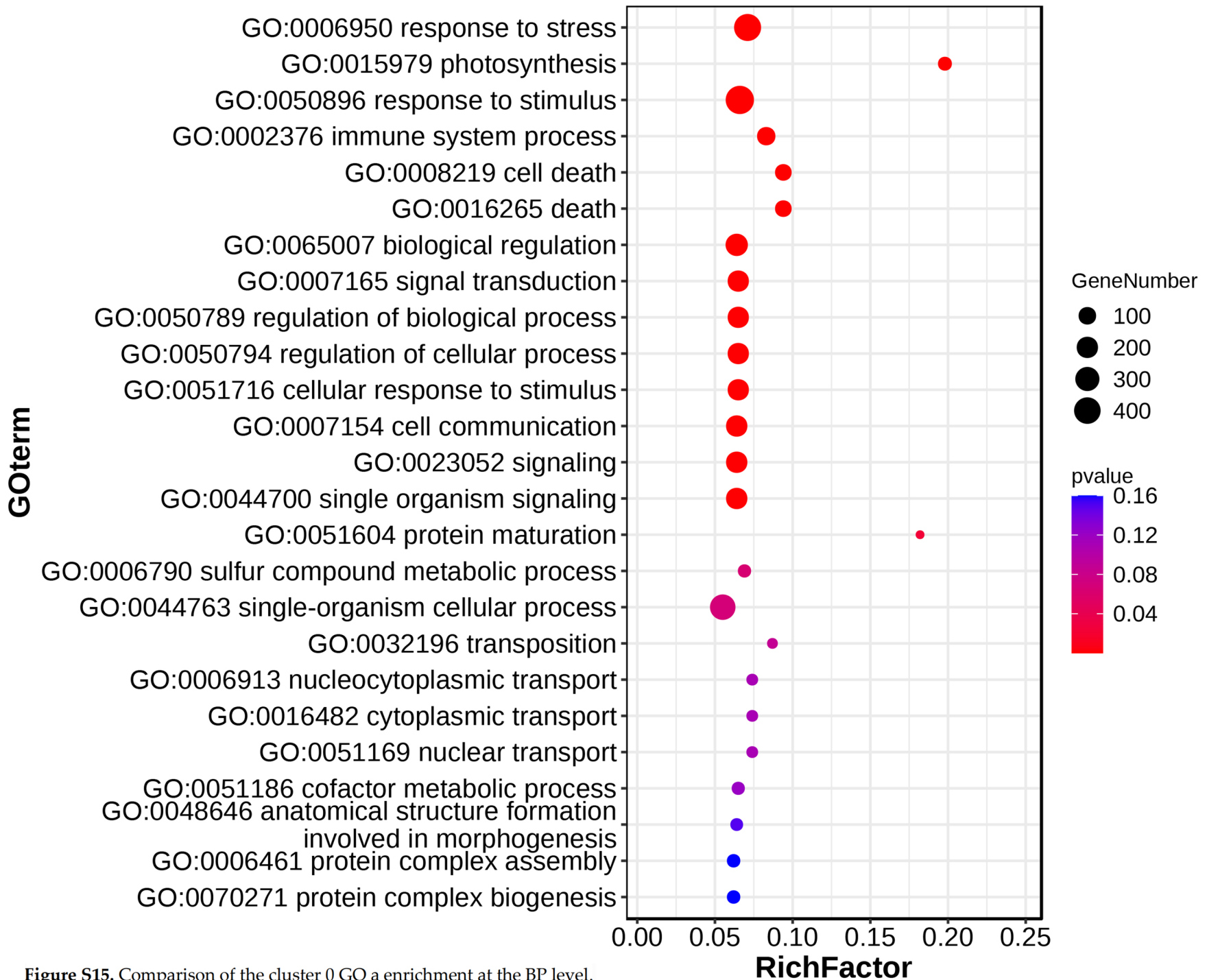

**Figure S15.** Comparison of the cluster 0 GO a enrichment at the BP level.

Supplement: Supplementary file 1 [file ijms-25-02427-s001.zip › Supplementary Figures/FigureS1 to S20.pdf/Figure S15. Comparison of the cluster 0 GO a enrichment at the BP level.pdf]

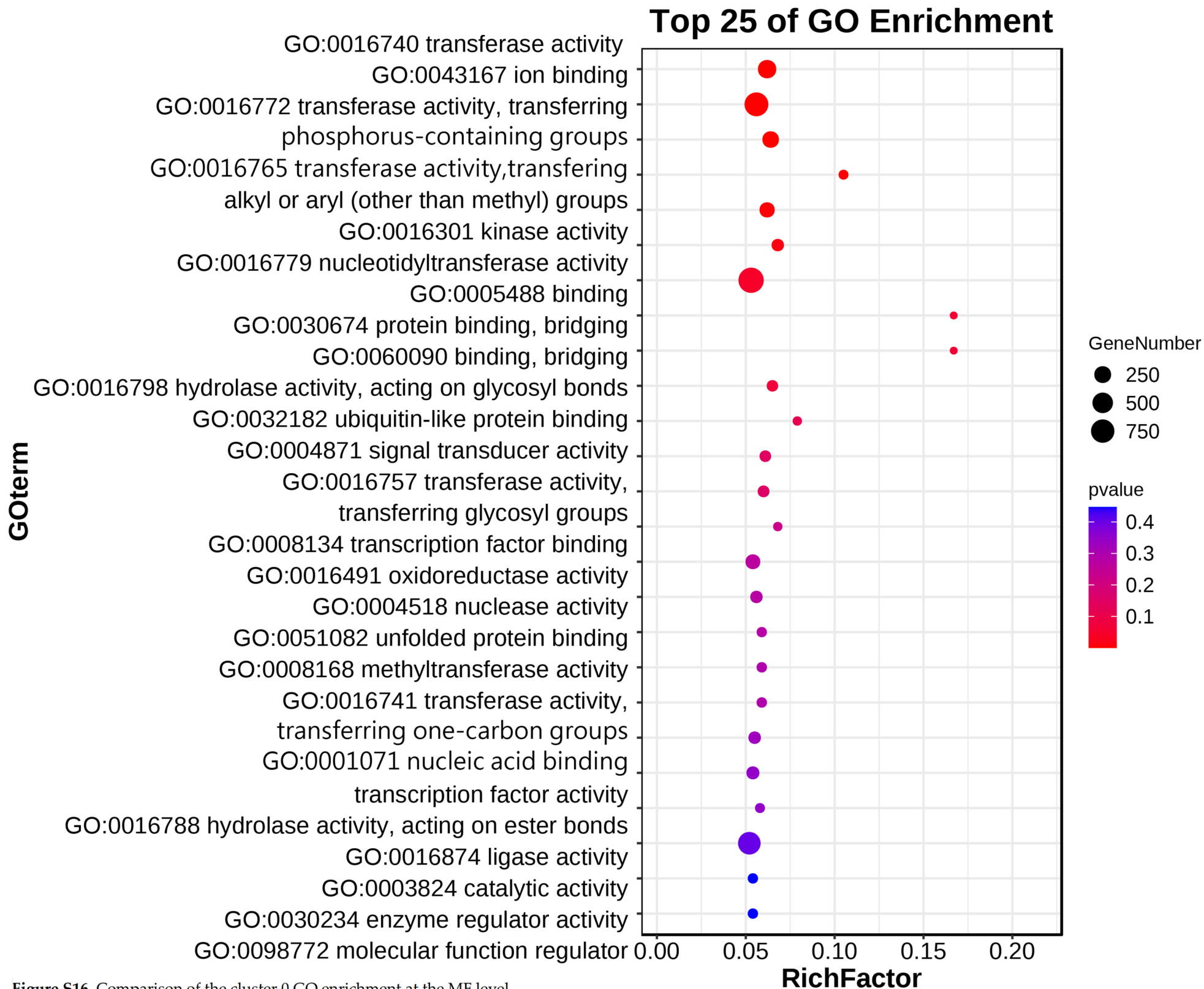

Figure S16. Comparison of the cluster 0 GO enrichment at the MF level.

Supplement: Supplementary file 1 [file ijms-25-02427-s001.zip › Supplementary Figures/FigureS1 to S20.pdf/Figure S16. Comparison of the cluster 0 GO enrichment at the MF level.pdf]

# Top 25 of GO Enrichment

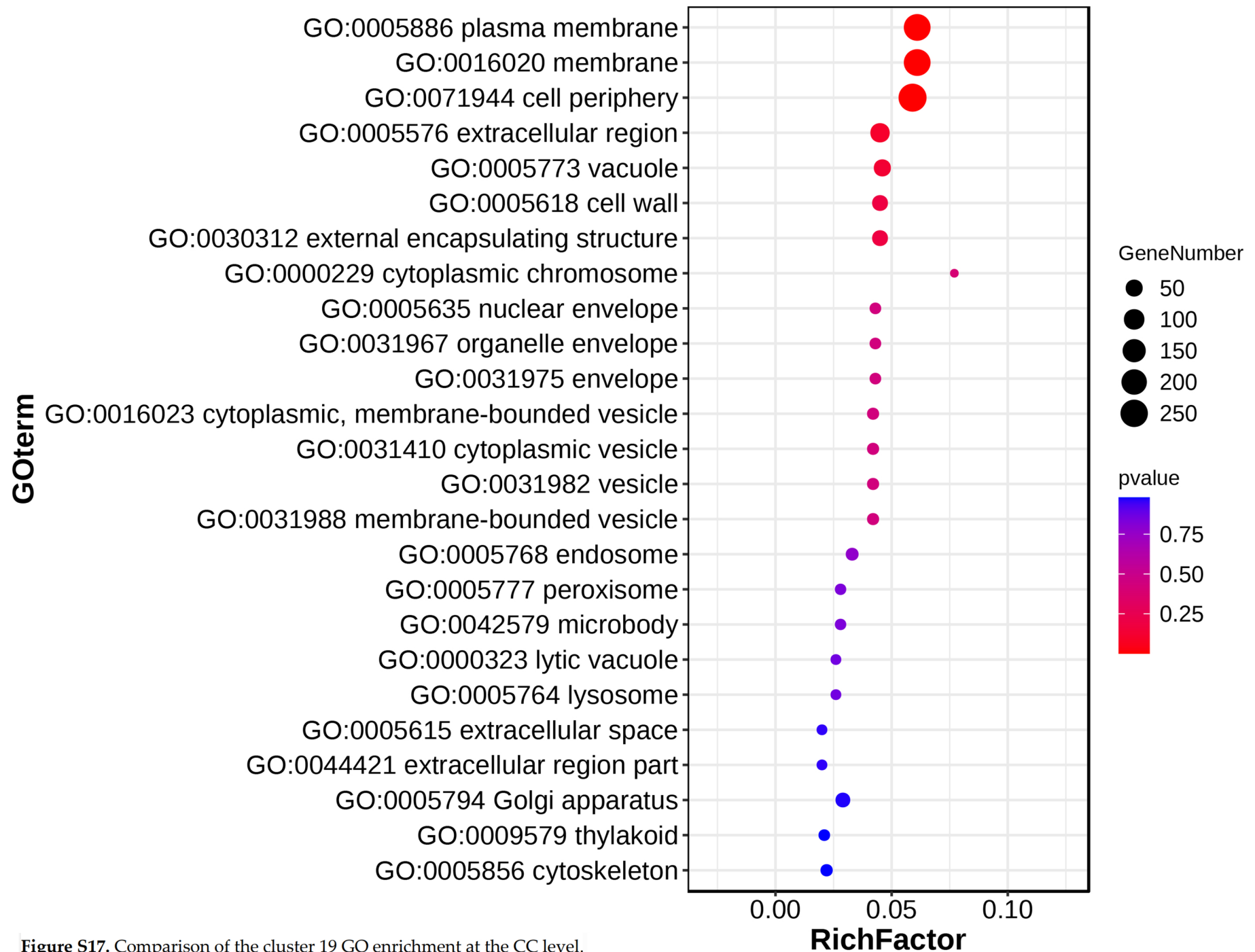

Figure S17. Comparison of the cluster 19 GO enrichment at the CC level.

Supplement: Supplementary file 1 [file ijms-25-02427-s001.zip › Supplementary Figures/FigureS1 to S20.pdf/Figure S17. Comparison of the cluster 19 GO enrichment at the CC level.pdf]

# Top 25 of GO Enrichment

GOterm

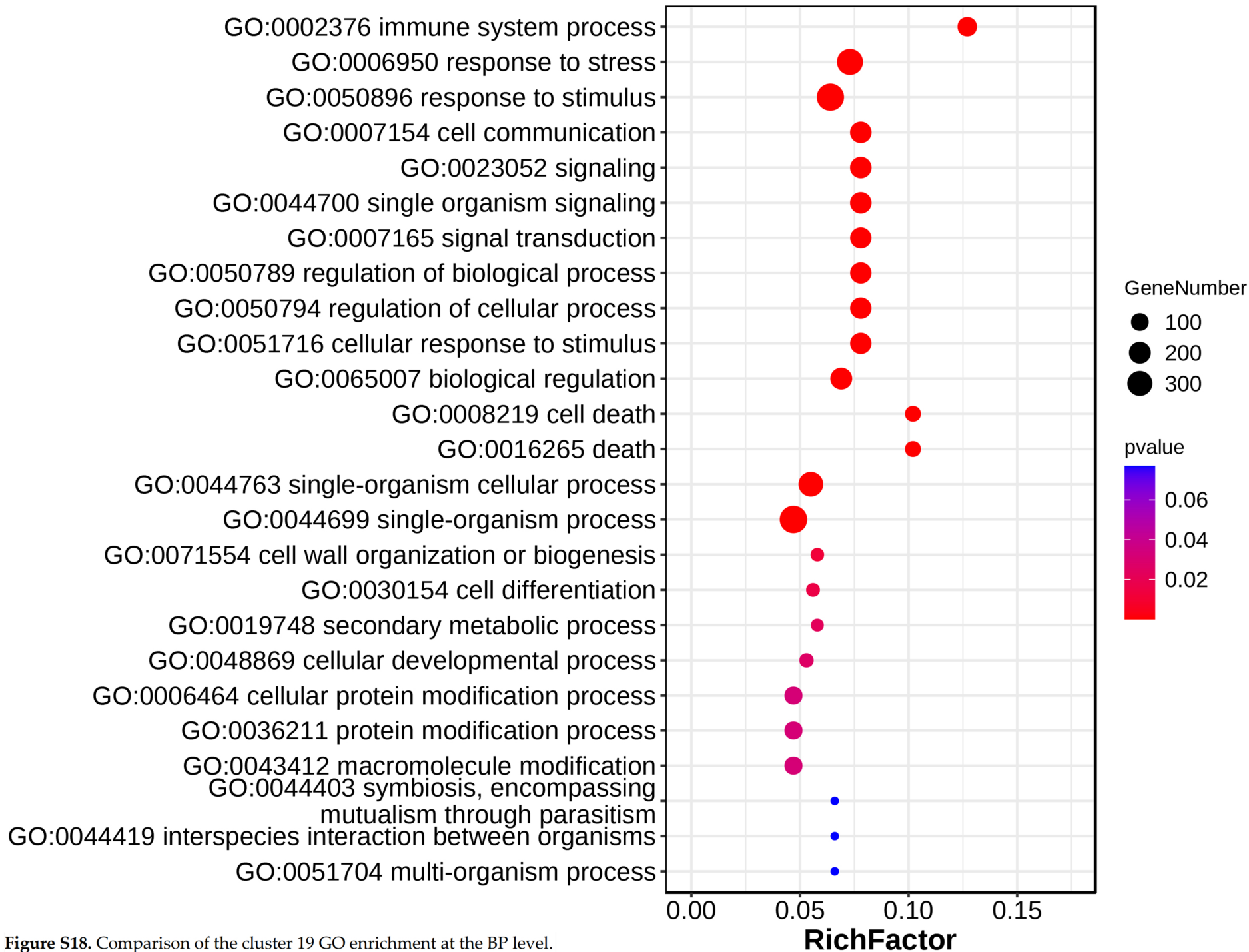

Figure S18. Comparison of the cluster 19 GO enrichment at the BP level.

Supplement: Supplementary file 1 [file ijms-25-02427-s001.zip › Supplementary Figures/FigureS1 to S20.pdf/Figure S18. Comparison of the cluster 19 GO enrichment at the BP level.pdf]

## Top 25 of GO Enrichment

GOterm

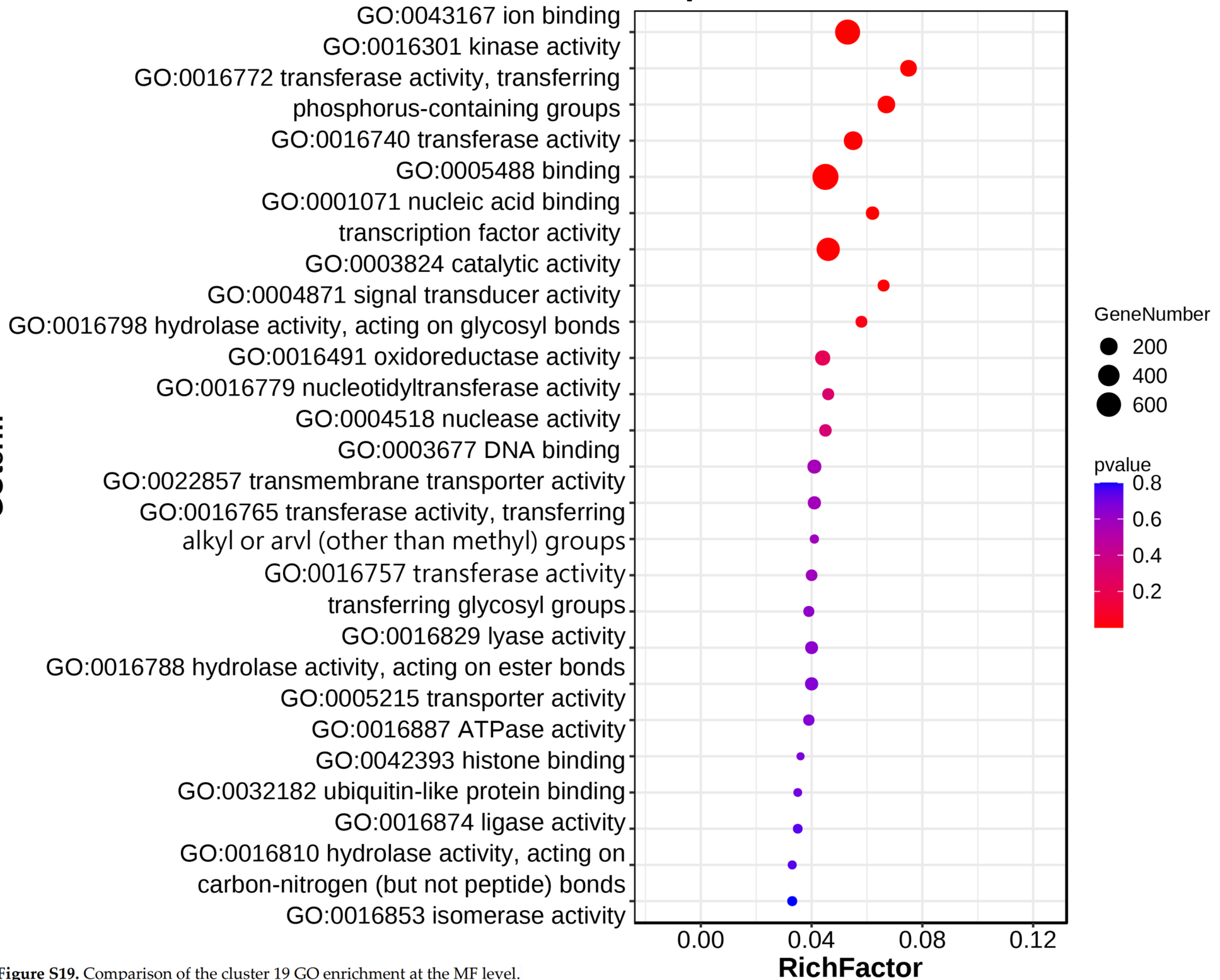

Figure S19. Comparison of the cluster 19 GO enrichment at the MF level.

Supplement: Supplementary file 1 [file ijms-25-02427-s001.zip › Supplementary Figures/FigureS1 to S20.pdf/Figure S19. Comparison of the cluster 19 GO enrichment at the MF level.pdf]

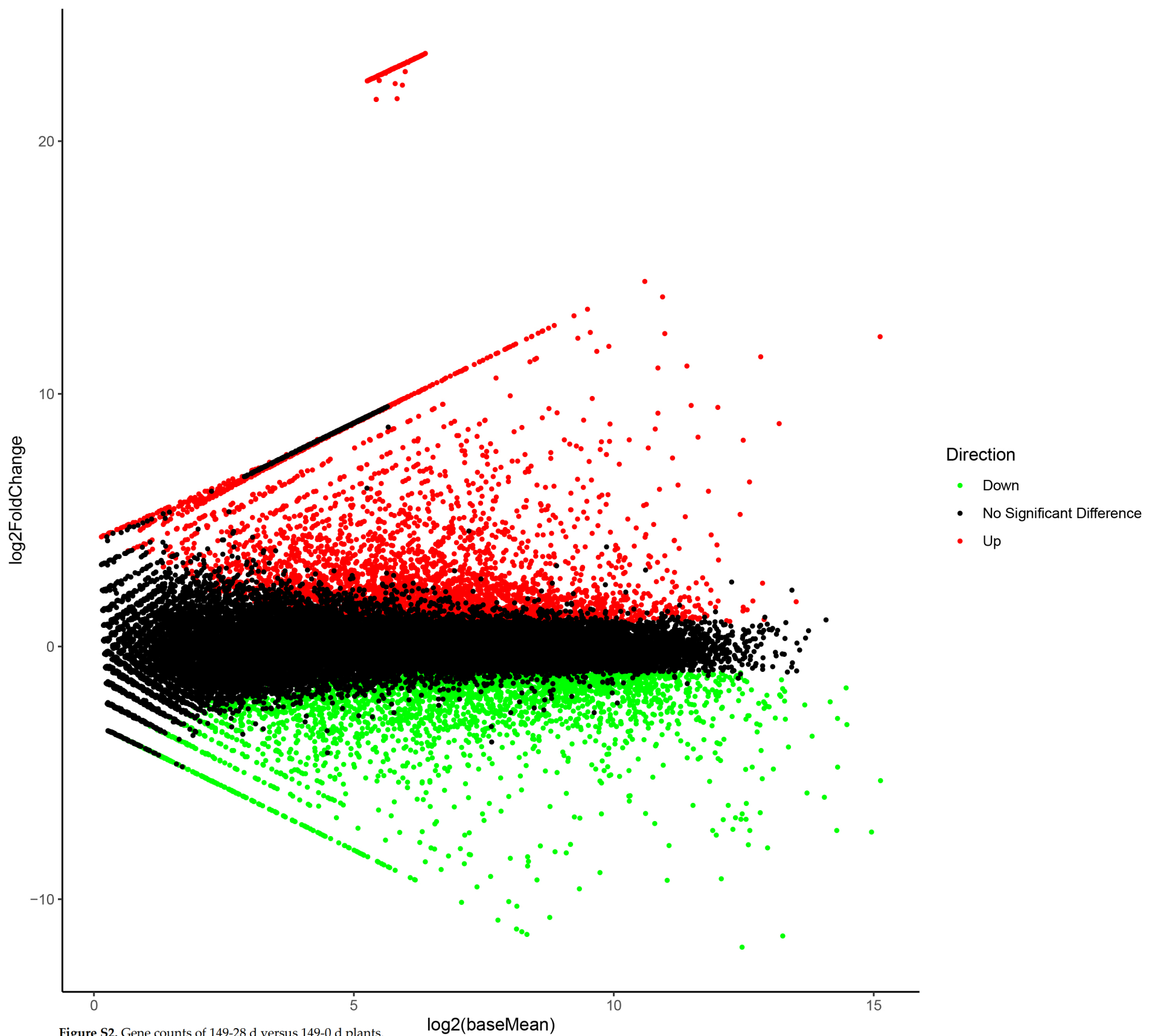

Figure S2. Gene counts of 149-28 d versus 149-0 d plants.

Supplement: Supplementary file 1 [file ijms-25-02427-s001.zip › Supplementary Figures/FigureS1 to S20.pdf/Figure S2. Gene counts of 149-28 d versus 149-0 d plants.pdf]

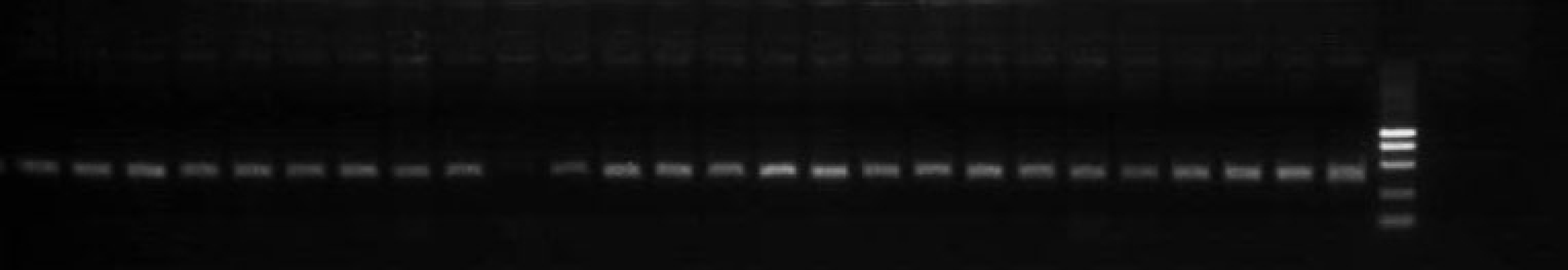

**Figure S20.** PCR and electrophoresis results

Supplement: Supplementary file 1 [file ijms-25-02427-s001.zip › Supplementary Figures/FigureS1 to S20.pdf/Figure S20. PCR and electrophoresis results.pdf]

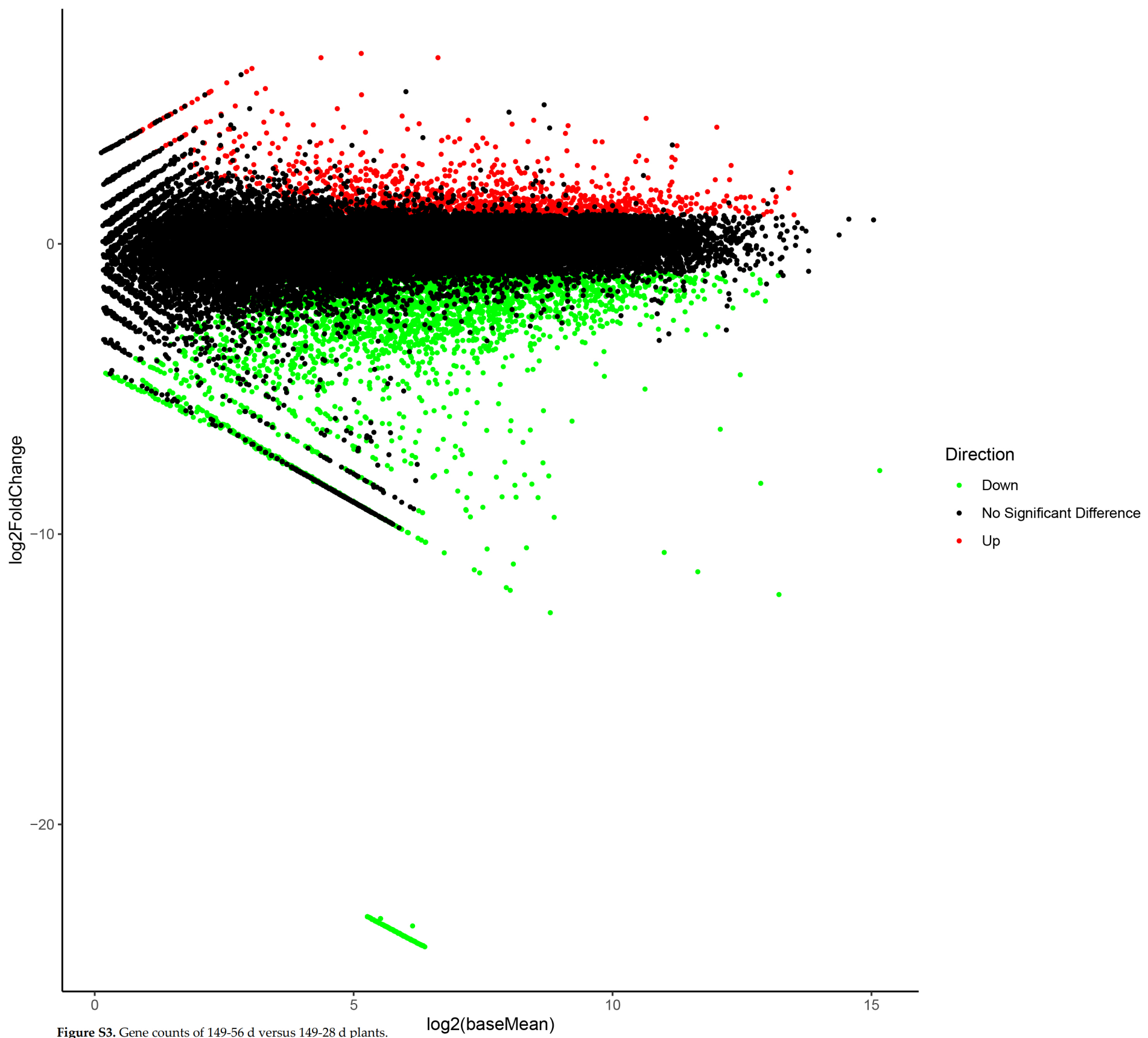

Figure S3. Gene counts of 149-56 d versus 149-28 d plants.

Supplement: Supplementary file 1 [file ijms-25-02427-s001.zip › Supplementary Figures/FigureS1 to S20.pdf/Figure S3. Gene counts of 149-56 d versus 149-28 d plants.pdf]

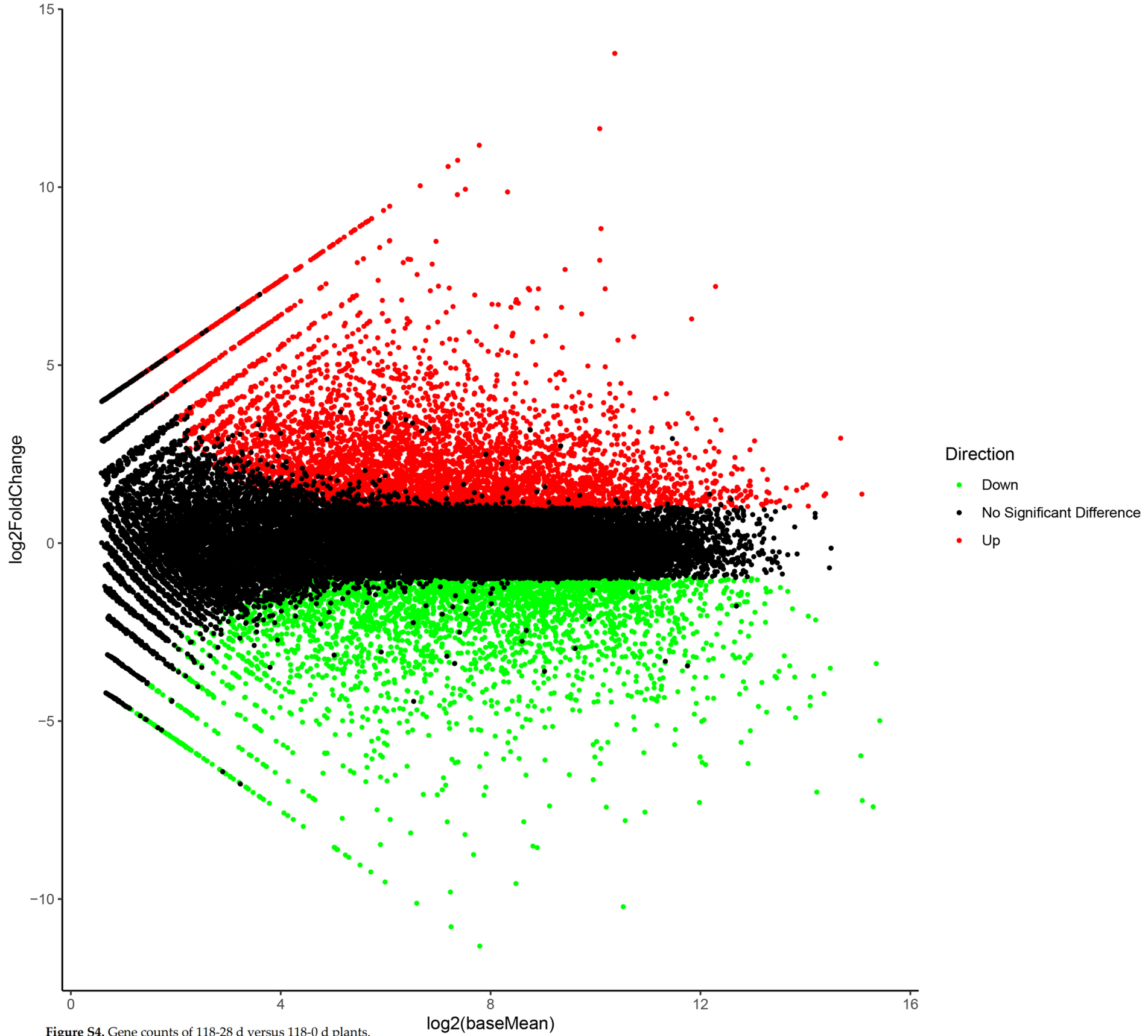

**Figure S4.** Gene counts of 118-28 d versus 118-0 d plants.

Supplement: Supplementary file 1 [file ijms-25-02427-s001.zip › Supplementary Figures/FigureS1 to S20.pdf/Figure S4. Gene counts of 118-28 d versus 118-0 d plants.pdf]

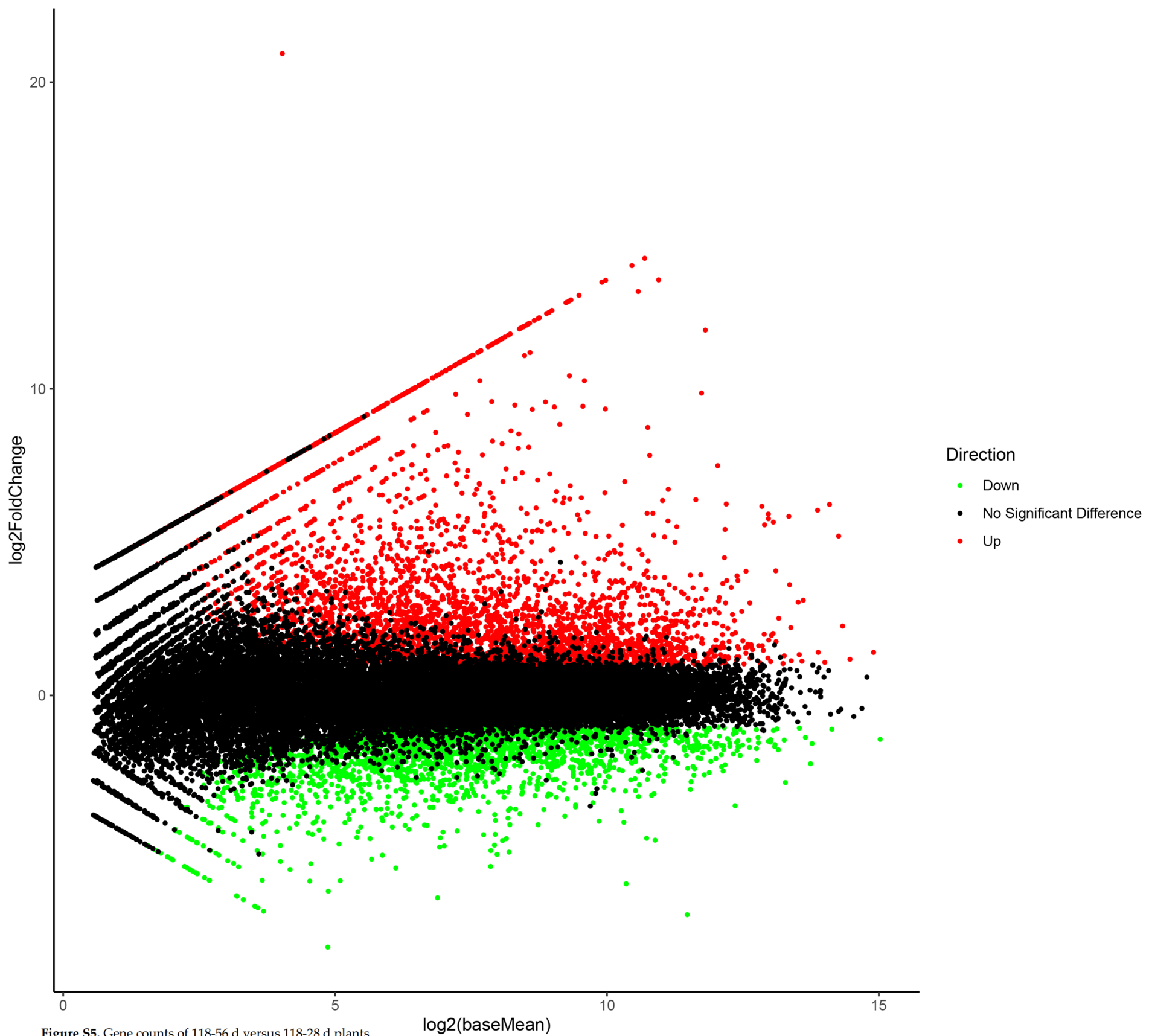

Figure S5. Gene counts of 118-56 d versus 118-28 d plants.

Supplement: Supplementary file 1 [file ijms-25-02427-s001.zip › Supplementary Figures/FigureS1 to S20.pdf/Figure S5. Gene counts of 118-56 d versus 118-28 d plants.pdf]

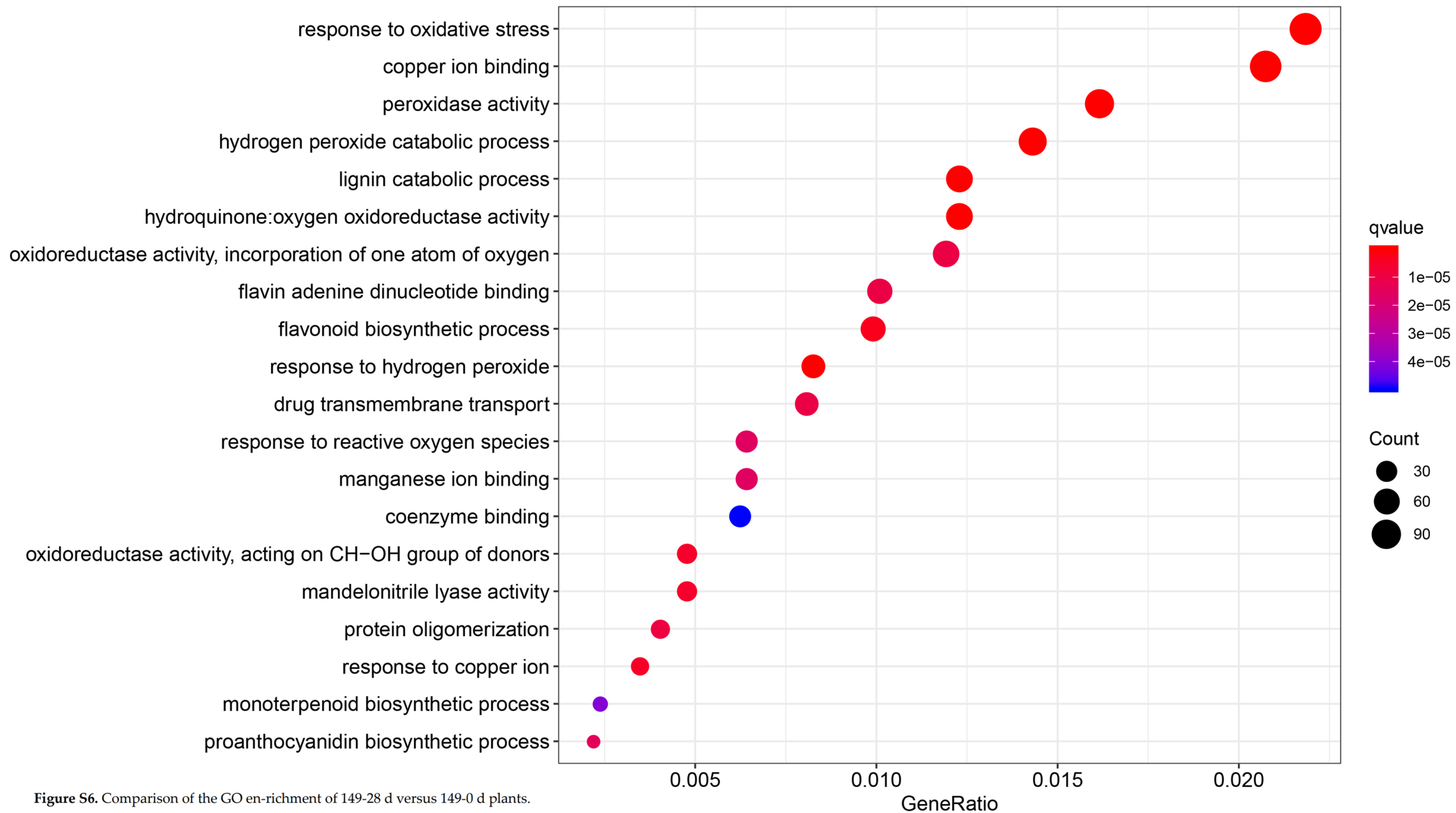

Supplement: Supplementary file 1 [file ijms-25-02427-s001.zip › Supplementary Figures/FigureS1 to S20.pdf/Figure S6. Comparison of the GO enrichment of 149-28 d versus 149-0 d plants.pdf]

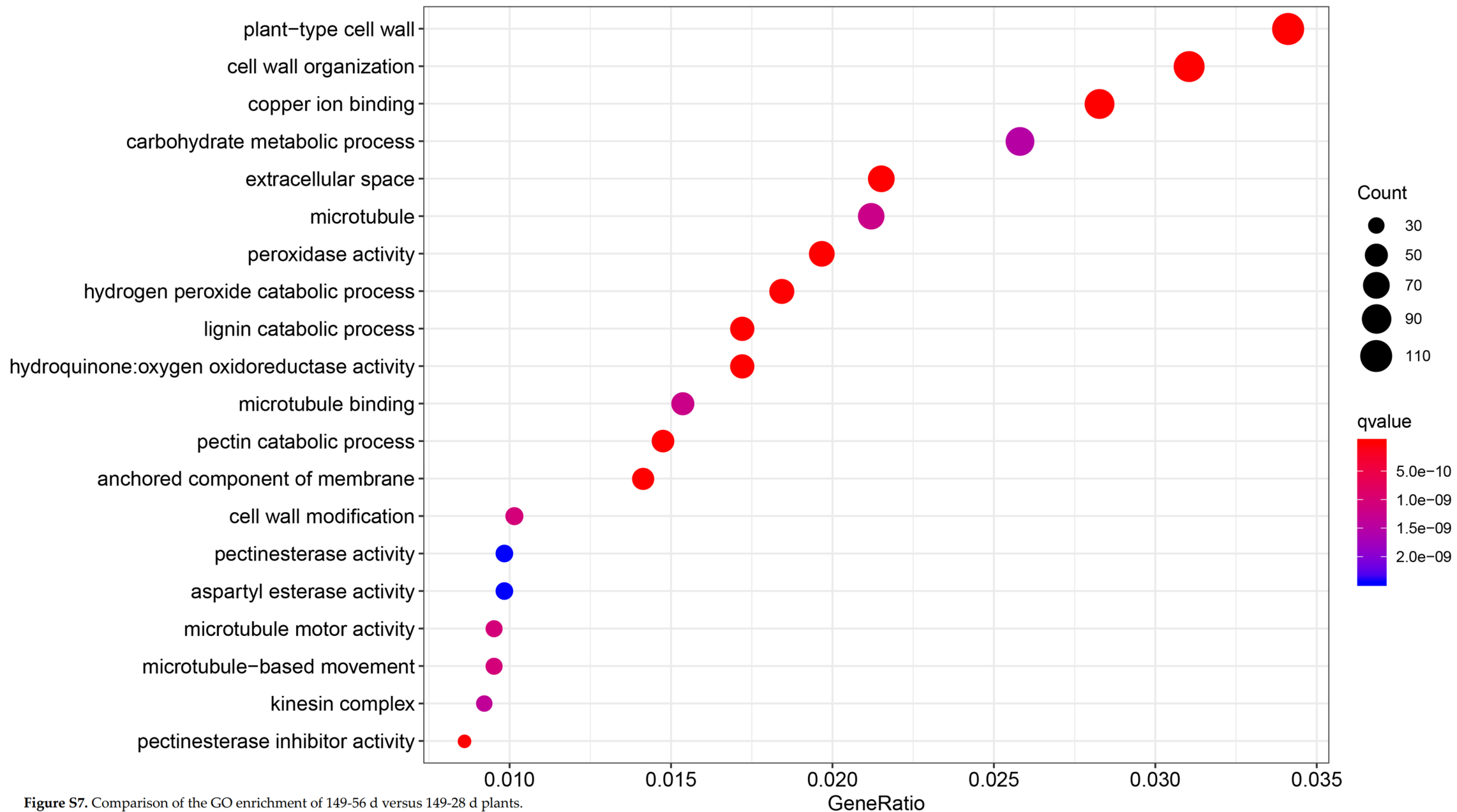

Supplement: Supplementary file 1 [file ijms-25-02427-s001.zip › Supplementary Figures/FigureS1 to S20.pdf/Figure S7. Comparison of the GO enrichment of 149-56 d versus 149-28 d plants.pdf]

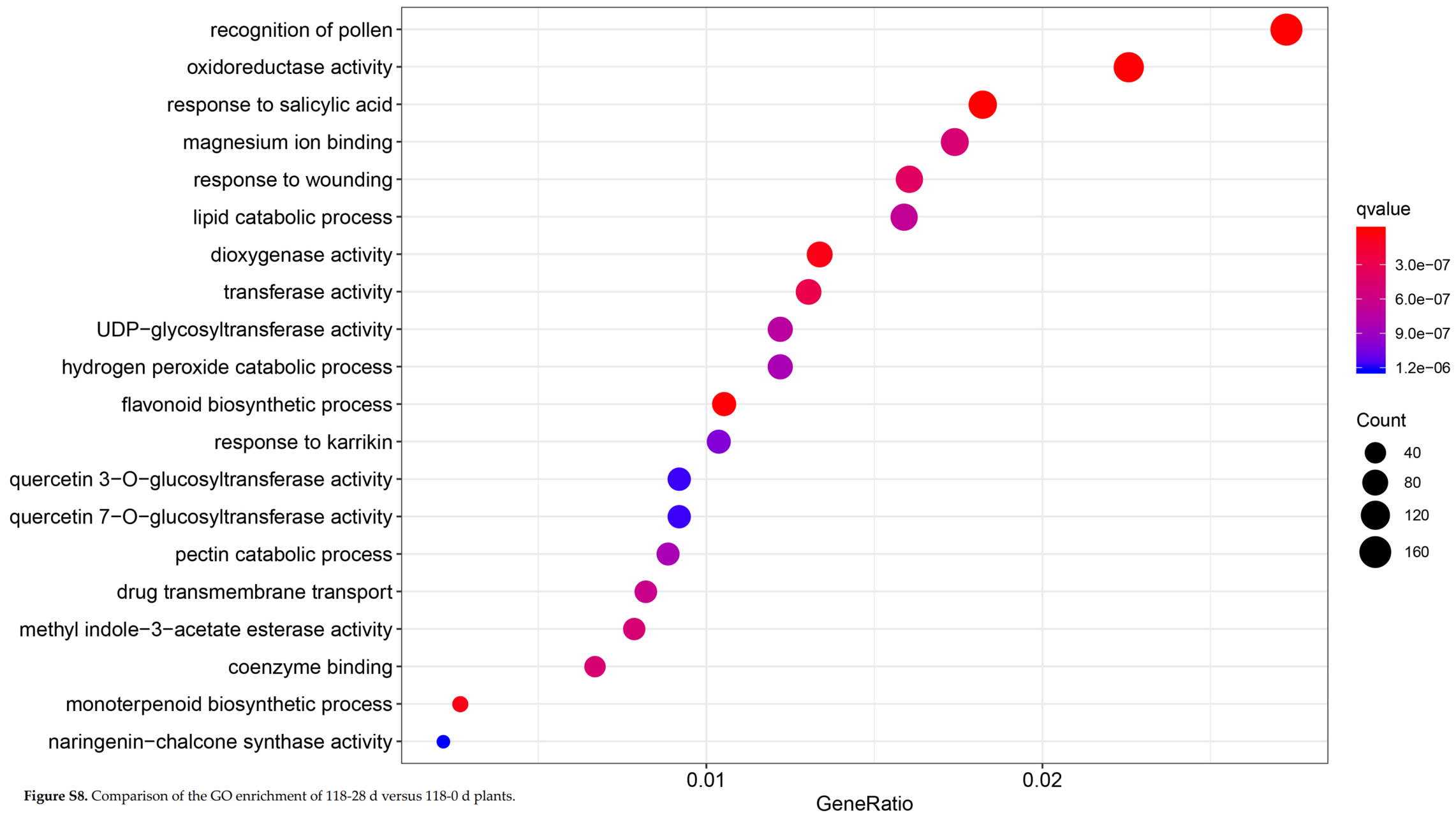

Supplement: Supplementary file 1 [file ijms-25-02427-s001.zip › Supplementary Figures/FigureS1 to S20.pdf/Figure S8. Comparison of the GO enrichment of 118-28 d versus 118-0 d plants.pdf]

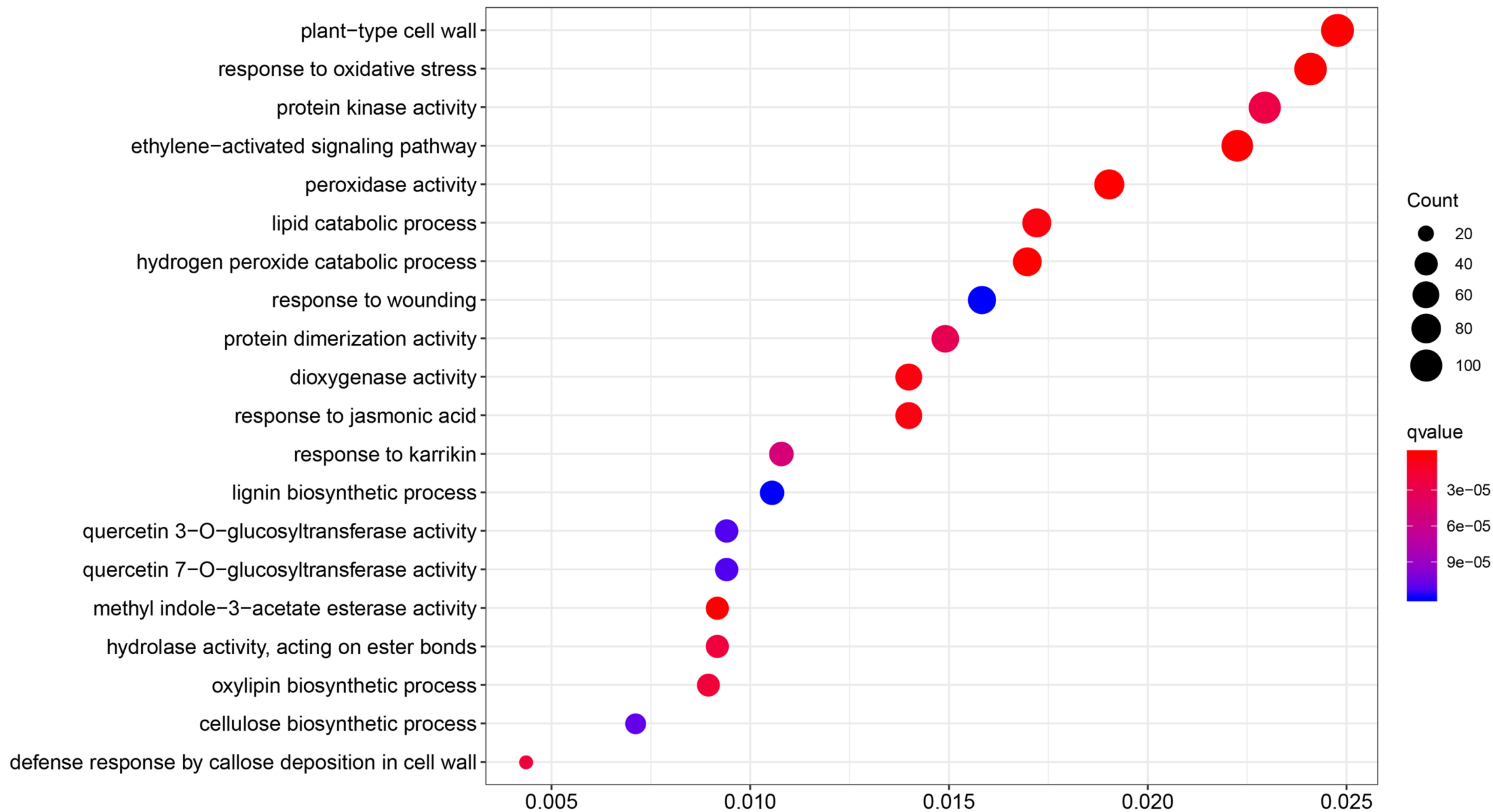

**Figure S9.** Comparison of the GO enrichment of 118-56 d versus 118-28 d plants.

Supplement: Supplementary file 1 [file ijms-25-02427-s001.zip › Supplementary Figures/FigureS1 to S20.pdf/Figure S9. Comparison of the GO enrichment of 118-56 d versus 118-28 d plants.pdf]
